# Effects of bifrontal transcranial direct current stimulation on brain glutamate levels and resting state connectivity: multimodal MRI data for the cathodal stimulation site

**DOI:** 10.1007/s00406-020-01177-0

**Published:** 2020-08-02

**Authors:** Eva Mezger, Boris-Stephan Rauchmann, Andre R. Brunoni, Lucia Bulubas, Axel Thielscher, Jana Werle, Matin Mortazavi, Temmuz Karali, Sophia Stöcklein, Birgit Ertl-Wagner, Stephan Goerigk, Frank Padberg, Daniel Keeser

**Affiliations:** 1 Department of Psychiatry and Psychotherapy, University Hospital, LMU Munich, Munich, Germany; 2 Department of Radiology, University Hospital LMU Munich, Munich, Germany; 3 Department of Psychiatry and Laboratory of Neurosciences (LIM-27), Institute of Psychiatry, University of Sao Paulo, Sao Paulo, Brazil; 4 International Max Planck Research School for Translational Psychiatry (IMPRS-TP), Munich, Germany; 5 Danish Research Centre for Magnetic Resonance, Centre for Functional and Diagnostic Imaging and Research, Copenhagen University Hospital Hvidovre, Hvidovre, Denmark; 6 Department of Health Technology, Technical University of Denmark, Lyngby, Denmark; 7 Department of Medical Imaging, The Hospital for Sick Children, University of Toronto, Toronto, Canada; 8 Hochschule Fresenius, University of Applied Sciences, Munich, Germany

**Keywords:** Magnetic resonance spectroscopy, Functional magnetic resonance imaging (fMRI), Electrical field modelling, Glutamate, GABA, Transcranial direct current stimulation (tDCS)

## Abstract

Transcranial direct current stimulation (tDCS) over prefrontal cortex (PFC) regions is currently proposed as therapeutic intervention for major depression and other psychiatric disorders. The in-depth mechanistic understanding of this bipolar and non-focal stimulation technique is still incomplete. In a pilot study, we investigated the effects of bifrontal stimulation on brain metabolite levels and resting state connectivity under the cathode using multiparametric MRI techniques and computational tDCS modeling. Within a double-blind cross-over design, 20 subjects (12 women, 23.7 ± 2 years) were randomized to active tDCS with standard bifrontal montage with the anode over the left dorsolateral prefrontal cortex (DLPFC) and the cathode over the right DLPFC. Magnetic resonance spectroscopy (MRS) was acquired before, during, and after prefrontal tDCS to quantify glutamate (Glu), Glu + glutamine (Glx) and gamma aminobutyric acid (GABA) concentration in these areas. Resting-state functional connectivity MRI (rsfcMRI) was acquired before and after the stimulation. The individual distribution of tDCS induced electric fields (efields) within the MRS voxel was computationally modelled using SimNIBS 2.0. There were no significant changes of Glu, Glx and GABA levels across conditions but marked differences in the course of Glu levels between female and male participants were observed. Further investigation yielded a significantly stronger Glu reduction after active compared to sham stimulation in female participants, but not in male participants. For rsfcMRI neither significant changes nor correlations with MRS data were observed. Exploratory analyses of the effect of efield intensity distribution on Glu changes showed distinct effects in different efield groups. Our findings are limited by the small sample size, but correspond to previously published results of cathodal tDCS. Future studies should address gender and efield intensity as moderators of tDCS induced effects.

**Electronic supplementary material:**

The online version of this article (10.1007/s00406-020-01177-0) contains supplementary material, which is available to authorized users.

## Introduction

Due to its safe and cost-effective profile, transcranial direct current stimulation (tDCS) of prefrontal cortex (PFC) regions represents a promising therapeutic approach in major depression (MD) and other psychiatric disorders [1–5]. The technique is based on the application of a weak direct current flowing between bipolar electrodes positioned over the head with an intensity of 1–2 mA for 5–30 min, for one or several days [6–8].

However, there is still an ongoing debate on the basic mechanisms of tDCS, such as the direction of its effects in terms of polarity, intensity, session duration and individual neuroanatomy [9–12]. Promising studies had shown that tDCS effects over the motor cortex were polarity-dependent, shifting neuronal resting membrane potentials either toward depolarization (close to the anode) or hyperpolarization (close to the cathode) [13–15]. Moreover, the orientation of neuronal layers [16, 17], anatomical differences between individuals, and the variability of brain states [18] may influence tDCS effects, as may current intensity and the precise electrode position, size and orientation [19–24].

When applying tDCS in different brain regions in healthy subjects various effects have been described, including changes in brain networks, assessed by resting-state functional connectivity magnetic resonance imaging (rsfcMRI) [25, 26], cognitive performance, measured via working memory tasks [27–31], and changes in brain metabolite and neurotransmitter levels, investigated via ^1^H-magnetic resonance spectroscopy (MRS) [28, 32–34]. In recent studies, rsfcMRI and computational modeling of the electrical field (efield) induced by tDCS in the brain have been included as additional tools to enhance the explanatory power, demonstrating an association of functional brain connectivity and/or efield strength with physiological changes [35–37].

Previous MRS studies mainly investigated tDCS over motor cortex regions [32, 34, 37–39], and very few MRS studies focused on tDCS of prefrontal regions [40, 41]. Bifrontal tDCS (anode: right dorsolateral PFC (right DLPFC; F4), cathode: left DLPFC (F3)) in gambling disorder increased GABA levels under the anode in the right DLPFC during stimulation. Another bifrontal tDCS montage (anode: F3, cathode: F4) in healthy participants increased prefrontal N-acetyl-aspartate (NAA) and striatal glutamate + glutamine (Glx) levels during and after stimulation. Combining on- and offline protocols for tDCS and MRS (i.e., MRS before, during and after stimulation) allows measuring dynamic effects of bifrontal tDCS. Similarly, adding another functional MR-based modality to MRS (i.e., rsfcMRI) can increase the explanatory power of these results as shown in a recent study [37]. In this pilot study, we investigated the effects of bifrontal tDCS on Glu, Glx and GABA levels in an MRS voxel close to the cathode over the right DLPFC before, during and after tDCS, expecting stimulation induced changes in metabolite concentration. In addition, we explored the impact of gender, efield distribution within the MRS voxel as well as rsfcMRI connectivity.

## Materials and methods

All subjects participated in a sham-controlled combined tDCS-MRS protocol and received active and sham tDCS in a double-blind cross-over design with randomized order.

The study was approved by the local ethics committee (Faculty of Medicine, Ludwig Maximilian University Munich, Munich, Germany). All participants provided written informed consent, and received financial compensation for participation.

### Participants

Twenty out of 25 recruited subjects (12 women [23.6 ± 2.0 years]/8 men [24.1 ± 2.0 years]; mean age of 23.7 ± 2.0 years) were analyzed. Five participants were excluded due to poor MRS data quality. Recruitment was performed via social networks (facebook.com) and postings at the University hospital. An online questionnaire was sent for screening to assess exclusion criteria, such as drug abuse, other psychiatric or neurological diseases and MRI contraindications (e.g., metals in/on the body, claustrophobia, pacemakers). In addition, a telephone interview was conducted to screen participants for psychiatric and neurological disorders, use of psychotropic medications, and unstable or severe physical health conditions. Participants were asked to abstain from alcohol the day before the measurement and to avoid caffeine on the day of the measurement. All participants were right-handed, as assessed by the Edinburgh Handedness Inventory [42].

### Experimental design

All participants underwent two combined tDCS-MRI sessions (approximately 2 h each) at the same time of the day with a minimum interval of 2 weeks between both sessions to avoid carry-over effects. Before and after tDCS-MRI measurements, positive and negative affect were assessed using the PANAS trait and state questionnaire (Positive And Negative Affect Schedule; [43]).

**Fig. 1:**
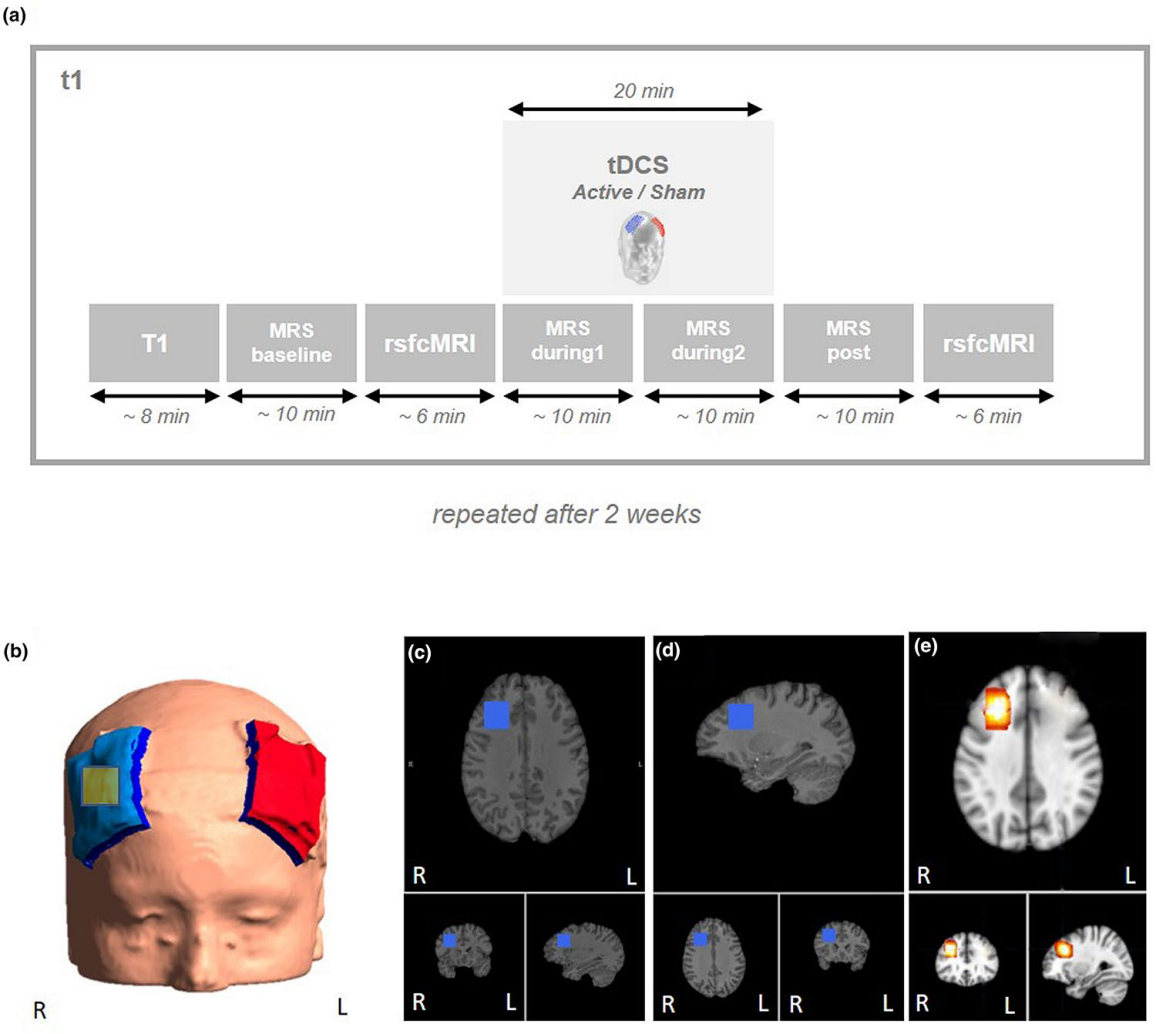
Study protocol. **a** Four 10-min intervals of MRS were measured before (baseline), during (during1, during2) and after (post) tDCS. MRS during1 was started 15 s after the beginning of tDCS. **b** Electrode positioning with the anode over the left DLPFC and the cathode over the right DLPFC. MRS region of interest (ROI) was placed under the cathodal electrode in the right DLPFC (yellow box). **c** Example of the MRS ROI in a male participant. **d** Example of the MRS ROI in a female participant. **e** Combined ROI of all participants (male and female); ROIs projected onto the MNI152 standard template

The study design is shown in Fig. 1; before stimulation, structural MRI scans (T1- and T2-weighted isotropic 3D sequences), an MRS sequence and a rsfcMRI sequence were acquired. Two separate MRS sequences were measured during stimulation, initiated after 15 s of tDCS, to compare early and late periods of tDCS. After stimulation, another set of MRS and rsfcMRI sequences was acquired. A total of four MRS acquisitions named baseline, during1, during2 and post were conducted. Baseline MRS was recorded before rsfcMRI to exclude possible effects of echo planar imaging (EPI) sequences on MRS [44]. Only after measuring the first 10 subjects in our study, we noticed that the MRS ROI was placed according to the neurological instead of the radiological convention. Thus, the respective MRS ROI was erroneously placed underneath the cathode. However, expecting effects of bifrontal tDCS in proximity to both electrodes we continued our tDCS-MRS protocol with the MRS ROI positioned over the right DLPFC. Such effects under both electrodes with an increase of Glu under the anode and a reduction under the cathode has been previously been demonstrated [32, 37, 45]. Future studies need to investigate further MRS ROI positions ideally applying multi-voxel MRS for localizing tDCS effects on metabolites.

### Transcranial direct current stimulation

TDCS was administered using an MR-certified Eldith stimulator MR (neuroConn, Ilmenau, Germany) via two saline-soaked surface sponge electrodes (5 × 7 cm^2^) placed over F3 (anode) and F4 (cathode; according to the international 10–20 system) corresponding to the left and right DLPFC. Active tDCS was administered in the scanner for 20 min at 2 mA intensity. Sham tDCS followed the built-in placebo-mode that limits stimulation to the 15 s ramp-up/ramp-down periods to mimic the somatosensory artefacts of active tDCS (skin warming, tingling). Blinding was assessed after every single session using a standardized questionnaire [46].

### Magnetic resonance imaging/magnetic resonance spectroscopy

All MRI scans were conducted on a 3 Tesla MRI scanner (Magnetom Skyra, Siemens Healthineers, Erlangen, Germany). A T1-weighted 3D structural magnetization-prepared rapid gradient-echo (MPRAGE) sequence with 176 layers of slices, slice thickness 0.8 mm^3^ isotropic voxels in sagittal orientation, repetition time (TR) = 1900 ms, echo time (TE) = 2.2 ms, flip angle (FA) of 9° and field of view (FoV) of 200 × 200 mm; and a T2-weighted 3D SPACE sequence with 160 slices, slice thickness 1.0 mm^3^ isotropic voxels, TR = 5000 ms, TE = 386 ms and FoV of 256 × 256 mm were acquired. Single-voxel spectroscopy with a MEGA PRESS sequence [https://www.cmrr.umn.edu/spectro/] [47] (TR = 2000 ms, TE = 68 ms, spectral bandwidth = 2000 Hz, 144 averages and editing pulses applied to the GABA spins at 1.9 ppm for refocusing only the GABA spins for the ON-signal, and at 7.5 ppm that do not affect any GABA spins for the OFF-signal) was acquired. As the GABA signal acquired at 68 ms is roughly 50% macromolecule, we refer to GABA as GABA + in the following sections. Voxel placement was performed by experienced MRS operators on the individual 3D-reconstructed T1-weighted images using the superior frontal sulcus, the lateral fissure, and the genu of the corpus callosum as anatomical landmarks (see supplemental information, Fig. 4).

For quantification of GABA + and Glx concentrations, the open source software Gannet 3.0 (http://www.gabamrs.com) was used, while for the Glu quantification off-spectra of the MEGA-PRESS sequence were analyzed in LCModel (Linear Combination Model, Version 2.1-1A; [48]. Results are presented in ratios to creatine (for more detailed information of processing steps please see supplemental information section 3.2).

Data with standard deviations (Cramér-Rao lower bounds) > 20% estimated by the LCModel and Gannet 3.0 were considered as poor quality and excluded from further analysis (five out of twenty-five). Tissue segmentation in the ROIs was performed using FSL FAST [49] to estimate the content of cerebrospinal fluid (CSF), grey matter (GM), and white matter (WM). The metabolite concentrations were corrected for partial CSF volume in the ROI [50].

### Resting state functional MRI connectivity

Sixteen out of twenty datasets were analyzed (4 datasets were excluded due to failed data processing). An EPI sequence with the following parameters was acquired: TR = 2000 ms; TE = 30 ms; flip FA = 80°; spatial resolution, 3 × 3 x 3 mm^3^; imaging matrix, 64 × 64; FoV = 192 × 192 mm^2^; number of slices 36; number of volumes, 250. The individual high-resolution MPRAGE data served as anatomical reference.

Pre-processing of the data was conducted using FSL 5.0.10 (https://fsl.fmrib.ox.ac.uk/fsl/fslwiki/), AFNI (https://afni.nimh.nih.gov/) version 18 and in-house scripts (Karali et al. [51, 52]; https://zenodo.org/record/3530897#.XfdzSWRKhPZ). For detailed information about pre- and post-processing of the data please see supplemental information, page 2 and 3.

### Computational modeling of electrical fields

Eighteen out of twenty datasets were analyzed (7 men, two datasets were excluded due to missing T2-weighted datasets). SimNIBS 2.0 (Stimulation in Non Invasive Brain Stimulation, https://www.simnibs.org/; Thielscher et al. [53]) was used to model the distribution and intensity of the efield. To generate the head models, T1- and T2-weighted MR images were fed into the ‘mri2mesh’ function of SimNIBS, that employs FreeSurfer and FSL functions to automatically segment the MR images into five tissue types (white matter, grey matter, skin, skull and cerebrospinal fluid) and subsequently creates individual tetrahedral head meshes from the segmentations [54–56]. Efield simulations are based on the Finite Element Method (FEM).

For twelve out of eighteen participants (4 men, six datasets were excluded due to failing transformation into volumetric space), the simulated data of the norm of the electric field was transformed into MNI standard volumetric space using a customized python script based on FSL and the following GitHub resource: https://github.com/ncullen93/mesh2nifti to extract the number of activated voxels thresholded at 0.3 in GM only, as a measure for efield strength.

Exploratively, we investigated the relationship between Glu changes and individual efield strength within the MRS ROI by dividing the sample of twelve participants into a “small” (*n* = 5) and a “large” (*n* = 7) efield group. The cut-off value defined to separate the two groups was the mean value of activated voxels of all subjects (6000 activated voxels). We hypothesize that a larger number of activated efield voxels in the volumetric space reflects a stronger potential response to electrical stimulation as indicated by the results of the simulation.

Exact number of data sets for each analysis is shown in the study flow chart in the supplemental information, Fig. 2.

### Statistical analysis

Statistical analyses were conducted using R (R Development Core Team, 2008, R: A language and environment for statistical computing. R Foundation for Statistical Computing, Vienna, Austria. ISBN 3-900051-07-0, https://www.r-project.org/). We performed linear mixed effects models (LMM) for repeated measurements to investigate differences in metabolite concentration change between the active and sham group incorporating four different time points (baseline, during1, during2 and post). Measurements were considered as nested within subjects. To control for gender effects, gender was included as a covariate to the LMM. Inter-individual differences in metabolite concentration at baseline were accounted for by including a random intercept term to the model. To account for subject-specific change rates, we tested if the inclusion of a random slope term would significantly improve the model fit. Nested models were compared using χ^2^-likelihood-ratio tests. Effect sizes reflecting the between-group differences in metabolite concentration change over time were calculated (Cohen’s *d*). Post-hoc analyses were planned (e.g., multiple comparisons between time points) if factors reached significance in the original LMM (two-sided *p* < 0.05).

#### Open science

All raw data and scripts will be available via OSF: https://osf.io/qgs57/.

## Results

### Behavioural data

PANAS scores before and after the stimulation were evaluated showing differences of the effect of time and PANAS scores for active and sham stimulation (*p* = 0.037). For both conditions PANAS scores were higher before (mean_active_ = 16.00 ± 9.6; mean_sham_ = 14.95 ± 7.1) compared to after (mean_active_ = 13.25 ± 8; mean_sham_ = 12.15 ± 8.6) the stimulation. For more detailed information on behavioral data please see supplemental information page 5 and 6.

tDCS effects on metabolite concentrations

**Fig. 2:**
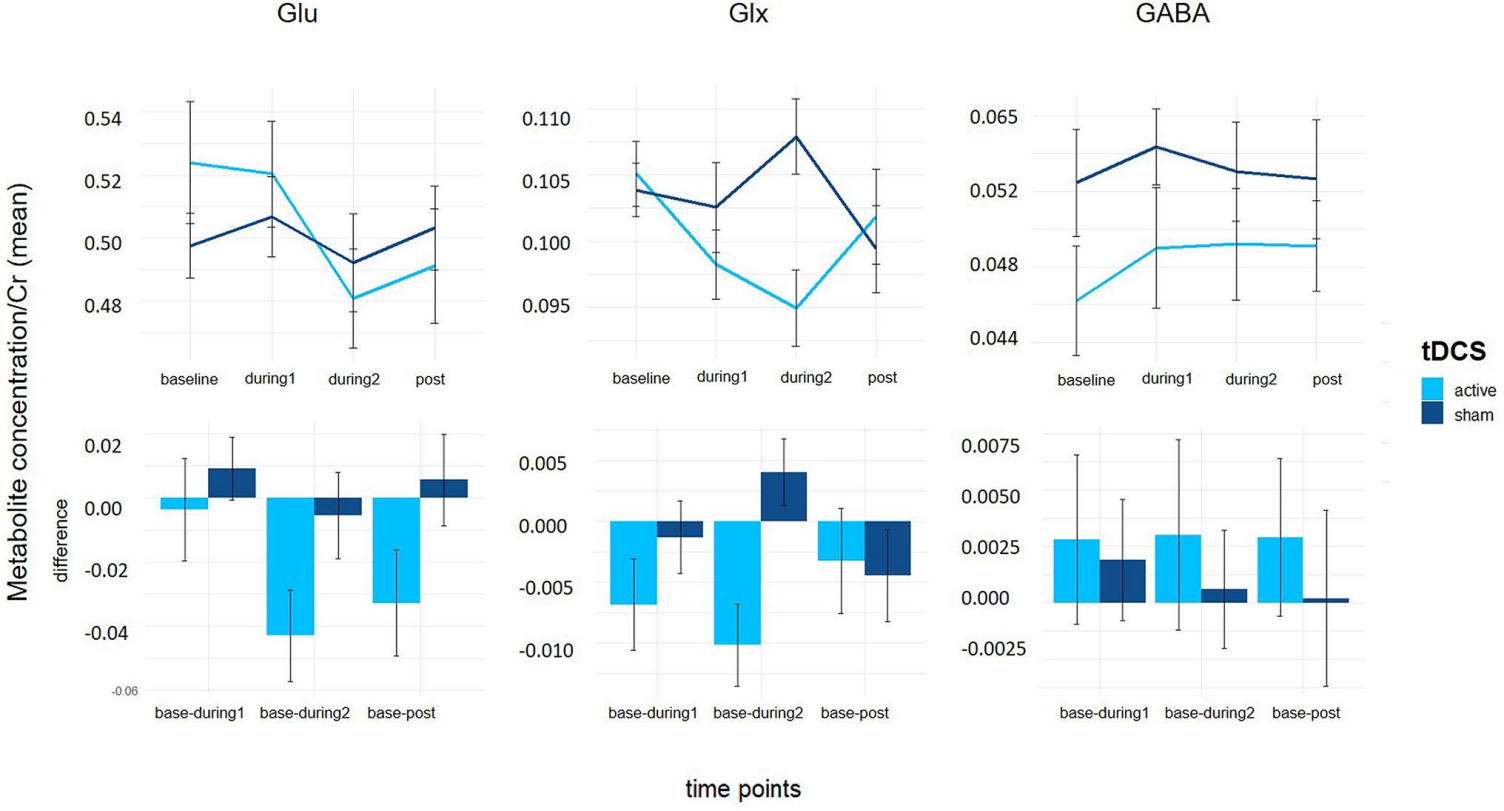
Glu, Glx and GABA + values from baseline to post stimulation of active and sham tDCS showing a significant reduction of Glu concentrations during active stimulation (see supplemental information, Table 1) and difference plots of metabolite changes to baseline concentrations. Error bars represent standard error of the mean (SEM). Glu, glutamate; Glx, glutamate & glutamine; GABA+, gamma aminobutyric acid (+ macromolecules)

We investigated changes of Glu, Glx and GABA + concentration over time in the two conditions (active, sham; see Fig. 2). Including a random slope term did not significantly improve model fit for all outcomes (Glu: χ^2^ = 0.03, *p* = 0.98; Glx: χ^2^ = 1.14, *p* = 0.57; GABA + : χ^2^ = 0.00, *p* = 1). Hence, a random intercept fixed slope solution was selected.

To control effects of Cr changes on Glu, GABA or Glx related effects, we also analyzed the NAA/Cr ratio. However, we did not detect any significant effects for time (*F*(1, 140) = 0.166, *p* = 0.685), condition (*F*(1, 140) = 0.065, *p* = 0.799) or the time*condition interaction (*F*(1, 140) = 0.037, *p* = 0.848).

### Effects of tDCS on Glu concentrations in the DLPFC

**Fig. 3:**
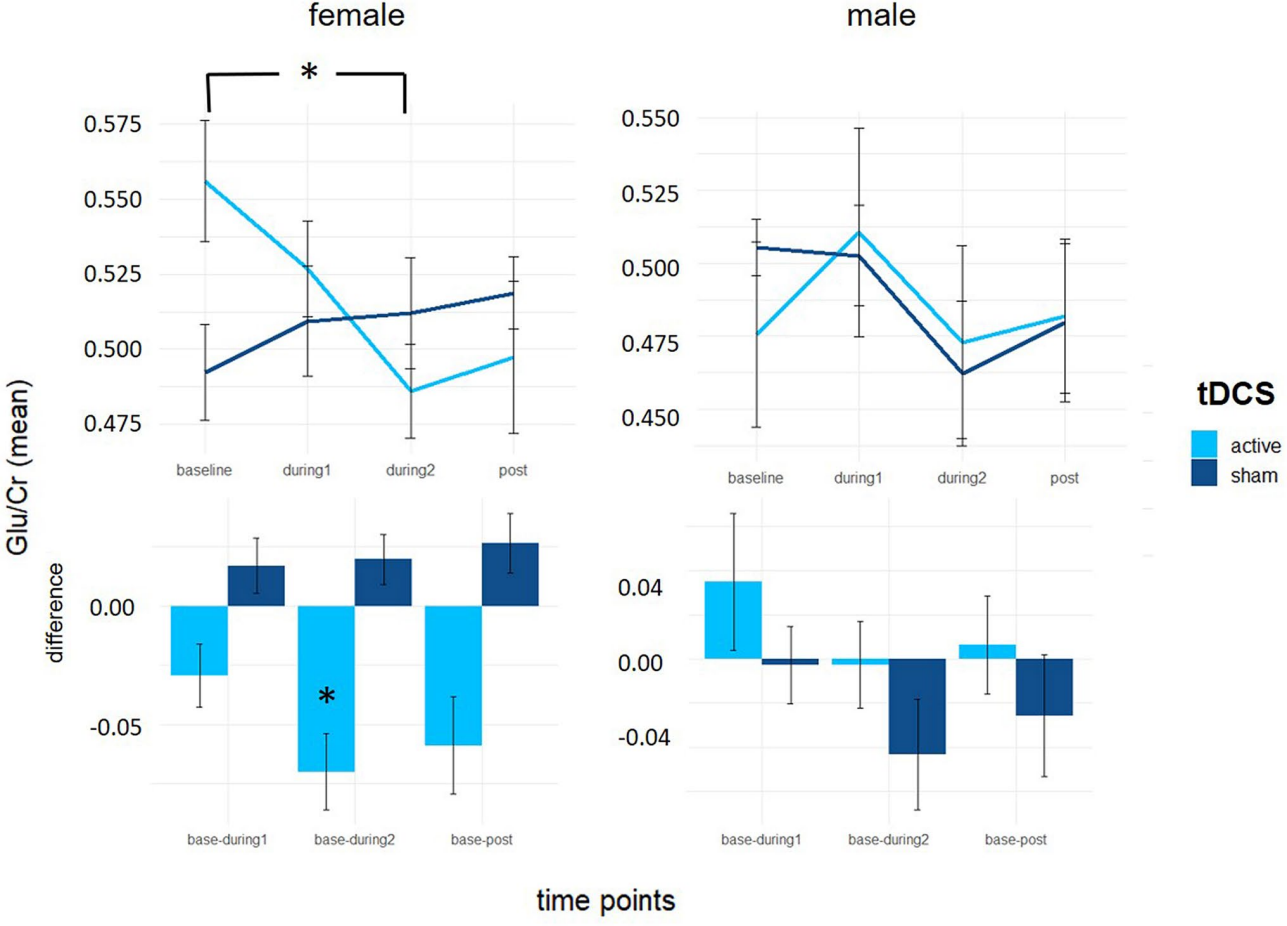
Glu concentration significantly decreased following bifrontal stimulation under the cathode in female but not in male participants. Error bars represent standard error of the mean (SEM); *p < 0.05. Glu, glutamate

While no significant effects were found for the factors time and condition, we observed a trend for the factor condition (*F*(_1, 140_) = 3.01, *p* = 0.085) and a trend for time*condition (*F*(_1, 140_) = 2.88, *p* = 0.092). Descriptive statistics showed a marked difference in male and female participants (Fig. 3). Therefore, we decided to additionally investigate how tDCS-induced changes differed with regard to gender as a model factor. No significant effects were found for the factors time (*F*(_1, 140_) = 2.67, *p* = 0.102) and time*condition (*F*(_1, 140_) = 1.48, *p* = 0.226) in the full sample (Fig. 2); however, the three-way interaction with gender indicated significant differences in tDCS-induced change of metabolite concentrations between male and female subjects (*F*_(1, 140)_ = 2.04, *p* = 0.017). Female subjects showed a significant reduction in Glu concentration in the active compared to the sham condition (*β* = 0.03 [0.01–0.05], *t*_(140)_ = 2.87, *p* = 0.004, *d* = 1.29 [0.41–2.17]), while male subjects did not (*β* = −0.01 [−0.03 to 0.02], *t*_(140)_ = 0.78, *p* = 0.440, *d* = 0.33 [− 0.50 to 1.16]) (see Fig. 3 and supplemental information Table 1). To determine at which time point tDCS-induced reduction in Glu (i.e., time*condition interactions) was at its strongest, Bonferroni-corrected LMM models were fit by consecutively including the next latest time point from baseline revealing a significant interaction for Glu change between baseline and the “during 2” time point (*β* =− 0.03 [− 0.05 to − 0.02], *t*_(12)_ = − 4.56, *p* = 0.004, *d* = 1.50 [0.86–2.15]) (see supplemental information, Table 2).

### Effects of tDCS on GABA + and Glx concentrations in the DLPFC

A significant effect of *gender* on GABA + concentration (*F*(_1, 140_) = 6.26, *p* = 0.014) was detected; however, no other significant interactions for the factors time (GABA: *F*(_1, 140_) = 0.28, *p* = 0.600; Glx: *F*(_1, 140_) = 1.57, *p* = 0.213), condition (GABA: *F*(_1, 140_) = 2.78, *p* = 0.100; Glx: *F*(_1, 140_) = 0.21, *p* = 0.645) and time*condition (GABA: *F*(_1, 140_) = 0.38, *p* = 0.540; Glx: *F*(_1, 140_) = 0.09, *p* = 0.764) were observed, neither for GABA + nor for Glx, even if examining gender separately (see supplemental information, Table 1).

### Effects of tDCS on resting-state functional connectivity

After active tDCS, rsfcMRI connectivity between the individual MRS ROI and whole brain showed an increase within the subgenual/subcallosal cortex (at trend level; cluster-corrected at 20 voxels, cluster: *x* = 2; *y* = 28; *z* = − 22 (21 voxel); log-*p* value = 1.0; FDR-corrected, see supplemental information Fig. 3). More detailed results are presented in supplemental information, page 8 and 9.

### Relationship between tDCS induced efields and brain metabolite changes

The individually modelled tDCS induced efields showed a high inter-individual variability of their distribution and peak intensities with efields widely spreading between the electrodes within the medial prefrontal cortex (see supplemental information, Fig. 1). Female participants showed significantly more activated voxels (mean = 7620, sd = 1676) compared to men (mean = 3141, sd = 1968) within the MRS ROI (*t*_(3)_ = 3.54, *p* = 0.038). We investigated whether the sex specific differences in tDCS induced Glu concentrations could also be found in subgroups of small and large efield. Therefore, we fit the same model with efield as grouping factor instead of gender. Based on the number of activated voxels of the transformed efield data into volumetric space, we defined groups of “small” and “large” efield. The “large” efield group revealed a trend towards stronger Glu reduction in the active compared to the sham condition (*β* = 0.02 [0.00–0.05], *t*_(84)_ = 1.69, *p* = 0.096, *d* = 1.23 [− 0.18 to 2.44]), while the “small” efield group did not (*β* = −0.01 [− 0.04 to 0.02], *t*_(84)_ = − 0.51, *p* = 0.613, *d* = 0.29 [− 0.84 to 1.43]) (see supplemental information, Table 3).

## Discussion

Our pilot study investigated the effects of a bifrontal tDCS protocol on Glu, Glx and GABA + levels close to the cathode over the right DLPFC. This tDCS protocol uses a standard montage which is commonly applied for the treatment of MD in clinical trials [57–60]. A reduction of Glu levels was observed for the active tDCS condition in a gender-dependent manner; however, no significant effects were found for Glx and GABA + concentration. For rsfcMRI, neither significant changes nor correlations with MRS data were observed except a trend (FDR-corrected, *p* < 0.1) for increased connectivity from the MRS-ROI to the subgenual/subcallosal cortex after active stimulation. Based on a computational model and individual MRI data, efields induced by tDCS were calculated to approximate the real efield distribution as a potential key parameter of individual tDCS dosing. Therefore, the study also aims to conceptually test a comprehensive multimodal neuroimaging approach (i.e., MRS, rsfcMRI and structural MRI based efield modeling), which to our knowledge has previously not been reported for prefrontal tDCS.

Prior studies combining tDCS and MRS have rather focused on M1 and SM1 and only very limited data are available for PFC regions. For motor and sensorimotor regions, a reduction of Glu levels was observed with several montages (i.e., cathode: left M1, anode: contralateral supraorbital ridge or cathode: left SM1, anode: right supraorbital region) [32, 37]. Thus, our results are in line with these previous findings supporting the central hypothesis of divergent effects of tDCS underneath cathode and anode, i.e., an inhibitory or excitatory action, respectively. Accordingly, increased Glu levels were detected in the right intraparietal sulcus after tDCS with the anode over the parietal cortex [45]. Opposite effects of tDCS on GABA levels were observed in prior studies, i.e., a reduction of GABA levels was detected in M1 (anode: left M1, cathode: contralateral supraorbital ridge) [32, 61, 62] and the occipital lobe (anode: occipital-temporal lobe, cathode: contralateral supraorbital ridge) [63].

Very few MRS studies to date have investigated the effects of prefrontal tDCS on brain metabolites. In the left DLPFC, NAA and striatal Glx levels were found to increase during tDCS with the anode over the left DLPFC (cathode over right DLPFC [40]) as well as GABA + concentrations during tDCS with the cathode over this region (anode over right DLPFC) [41]. The current study did not show such a modulation of GABA + levels. However, this negative finding should be interpreted with caution due to the limited sample size and a potentially large beta error.

Changes of Glu levels during and after tDCS as observed in the current study are hypothesized to emerge from a direct effect on neural firing rates and NMDA receptor dependent, long-lasting synaptic potentiation in animal models [64, 65]. However, metabolic changes in distinct MRS ROIs may also be induced transsynaptically through other brain regions functionally connected to the ROI, e.g., tDCS of the DLPFC may modulate metabolite concentrations in medial prefrontal regions [66]. In addition, it is not clear how PFC and motor regions actually differ in their functional response to tDCS with respect to Glu and GABA + levels, since both macro- and microconnectivity as well as regional neurotransmission differ largely across brain regions. In this pilot project, we observed Glu changes during, but not after tDCS as shown in previous studies [29, 34, 37, 38, 61, 67].

The gender-dependent Glu reduction in our study may be discussed in the light of gender-specific differences in metabolite levels (mainly Glu, Glx, GABA + and NAA) as previously reported; however, findings in prior studies were not fully consistent [68–70]. Numerous factors may theoretically contribute to a gender-dependence of tDCS effects on MRS measures, e.g., differences in brain metabolism and structure or hormonal status [71–73]. Though we found significant differences between male and female participants in their response to prefrontal tDCS, we have to consider that this effect may as well be due to the responder vs non-responder distribution in this small sample. Previous studies showed marked inter-individual differences between subjects in terms of their response to tDCS [25, 77]. This is an important factor and should be addressed in future studies by including additional measures (e.g., behavioral or neurophysiological information) which allows to classify responders vs non-responders. Moreover, future studies should survey gender-specific parameters to systematically investigate the role of these factors.

Having observed a marked difference in gender-specific efield intensities, we were interested in the question whether effects of tDCS may be related to individual efield intensities as shown in a previous study [37]. We observed similar differences in Glu concentrations between participants with “small” and “large” efields as defined by below or above the mean value of activated voxels for all subjects. Although these results are preliminary, the relationship between efield intensity and tDCS effects on metabolite concentrations may be relevant and should be further investigated and may be an avenue for establishing dose–response relationships for tDCS. The inter-individual variation of efields beyond the MRS ROI converges with previous evidence of a marked inter-individual variability in terms of efield intensities and their distribution [74, 75], and raises the question at which brain regions bifrontal montages actually exert their effects. In contrast to our study, Antonenko et al. [37] investigated normal components of efield strength (i.e., calculation of the efield including information about the efield entering or leaving the surface which is only available in SimNIBS 2.1) to address polarity effects of the stimulation, showing peaks of efield intensities at the stimulation site which may provide a superior approach for analyzing target specificity.

Offline rsfcMRI showed an increased network connectivity at a trend level (FDR-corrected, *p* < 0.1) from the right DLPFC ROI to the midline/right subgenual region, underscoring the importance of connectivity between both regions for network effects of prefrontal tDCS [2, 25, 76]. However, this was not associated with changes in Glu concentrations. We did not find differences between active and sham tDCS for within-ROI connectivity or ICA networks, though other studies showed this effect [18, 25, 37, 77]. The negative result may be explained by the small sample size and future studies with larger samples should address this issue again. Despite its relevance as a conceptual pilot project, our study has obvious limitations that need to be considered when interpreting the data. As said, the sample size is critically low, which is even more problematic at the subgroup level (defined by gender or efield parameters); however, it is comparable with sample sizes in previous tDCS-MRS studies (e.g., *N* = 17 in Hone-Blanchet et al. [40], *N* = 12 in Bachtiar et al. [38], *N* = 20 in Dwyer et al. [78], *N* = 24 in Antonenko et al. [37]). Thus, larger trials are clearly missing in the field. Another issue is that study protocols are critically diverse hampering a direct comparison of our results with previous findings by the large variation in tDCS and imaging methods including different on- and offline designs. In contrast to offline tDCS, MRS protocols, which were applied in the majority of studies [32, 35, 40, 45, 61, 63], combined on- and offline protocols as used here could be very informative regarding dynamic changes of brain metabolites, but were used in very few studies [38, 40, 61]. There is also a marked heterogeneity of tDCS targets and parameters (i.e., stimulation intensity and duration). Stimulation intensity varied between 1 and 2 mA and duration between 10 and 30 min in earlier MRS studies [14, 32, 35, 40, 45, 61, 63]. Here, we applied 2 mA intensity with a bifrontal montage (anode F3, cathode F4), since such protocols were used in previous studies in MDD and schizophrenia [2, 8, 60, 79, 80].

A specific restriction in using MRS for experimental research on tDCS is the key limitation of single voxel MRS, which does not allow to investigate tDCS effects for several regions in parallel. This is particularly critical in bipolar tDCS montages where already two regions are of main interest, and neither electrode can be a priori defined as inactive or reference. As a solution, multi-voxel MRS should be established in future tDCS studies to measure stimulation effects across several brain regions at the same time [81–83].

A final limitation is the investigation of only one stimulation montage, in which specific questions such as the relevance of electrode positions or current directions cannot be addressed [18, 84]. MRS data for ROIs close to anodal [14, 85] as well as cathodal cortical targets [27, 32] are available, and differences in baseline metabolite concentrations are still in the range of known variability [86–88].

## Conclusion

To the best of our knowledge, this is the first study investigating prefrontal tDCS in a combined on– and offline approach with the anode over the left DLPFC and the cathode over the right DLPFC using multimodal neuroimaging including MRS and MRI based efield modeling. Our main focus was feasibility and we observed that a standard bifrontal tDCS montage (anode—F3, cathode—F4), as is common in therapeutic trials, led to a reduction of Glu levels in the MRS voxel close to the cathode in female but not in male participants. Computational modelling of tDCS-induced efields based on individual MRI data shows a large inter-individual variation in efield intensity distribution, and preliminary evidence suggests that effects on Glu levels may vary with efield strength. As a conclusion, we support the idea to further develop the combined approach using MRS (ideally multi-voxel MRS), rsfcMRI and individual MRI based efield modeling for investigating the effects of current tDCS protocols on brain metabolites [37].

## Electronic supplementary material

Below is the link to the electronic supplementary material.

Supplementary material 1 (DOCX 3003 kb)
