# Supplementary material for: Effects of bifrontal transcranial direct current stimulation on brain glutamate levels and resting state connectivity: multimodal MRI data for the cathodal stimulation site
Source: Eur Arch Psychiatry Clin Neurosci. 2020 Aug 2;271(1):111–22. doi: 10.1007/s00406-020-01177-0 (PMC7867555; doi:10.1007/s00406-020-01177-0)
Supplement: Supplementary file 1 — Supplementary material 1 (DOCX 3003 kb) [file 406_2020_1177_MOESM1_ESM.docx]

Title:

Effects of bifrontal transcranial direct current stimulation on brain glutamate levels and resting state connectivity: Multimodal MRI data for the cathodal stimulation site.

Authors:

Eva Mezger^1*^, Boris-Stephan Rauchmann^1,2^, Andre R Brunoni^1,3^, Lucia Bulubas^1,4^, Axel Thielscher^5,6^, Jana Werle^1^, Matin Mortazavi^1^, Temmuz Karali^1^, Sophia Stöcklein^2^, Birgit Ertl-Wagner^7^, Stephan Goerigk^1,8^, Frank Padberg^1*^, Daniel Keeser^1,2^*

^1^*Department of Psychiatry and Psychotherapy, University Hospital, LMU Munich, Munich, Germany*

^2^*Department of Radiology, University Hospital LMU Munich, Germany*

*^3^Department of Psychiatry and Laboratory of Neurosciences (LIM-27), Institute of Psychiatry, University of Sao Paulo, Sao Paulo, Brazil*

*^4^International Max Planck Research School for Translational Psychiatry (IMPRS-TP), Munich, Germany*

*^5^Danish Research Centre for Magnetic Resonance, Centre for Functional and Diagnostic Imaging and Research, Copenhagen University Hospital Hvidovre*

*^6^Technical University of Denmark, Department of Health Technology*

*^7^Department of Medical Imaging, The Hospital for Sick Children, University of Toronto, Toronto, Canada*

*^8^Hochschule Fresenius, University of Applied Sciences, Munich, Germany*

**these authors equally contributed to this work.*

Corresponding author

Dr. Daniel Keeser

Email: [daniel.keeser@med.uni-muenchen.de](mailto:daniel.keeser@med.uni-muenchen.de)

Phone: +49 89 4400 55755

Supplemental Information

002 005 006 007 008 009 010 011 012 015


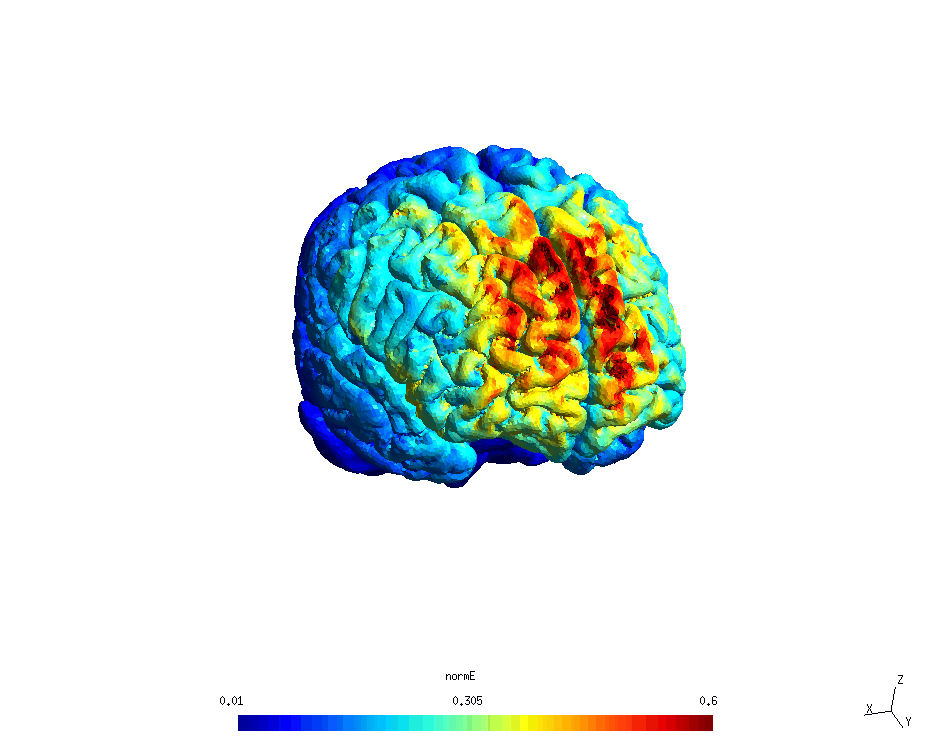

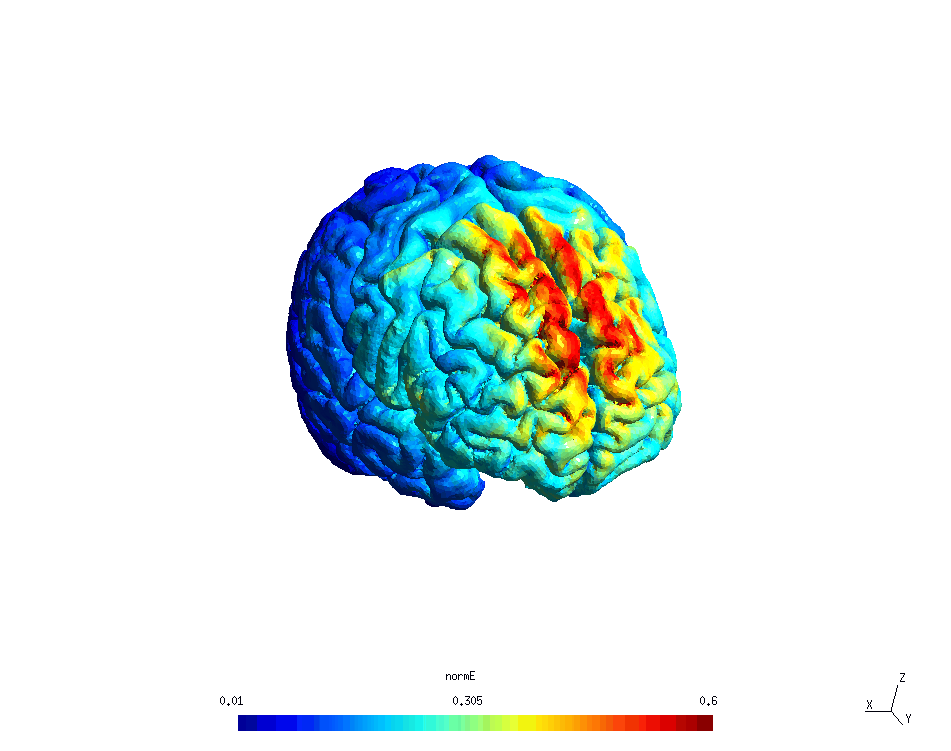

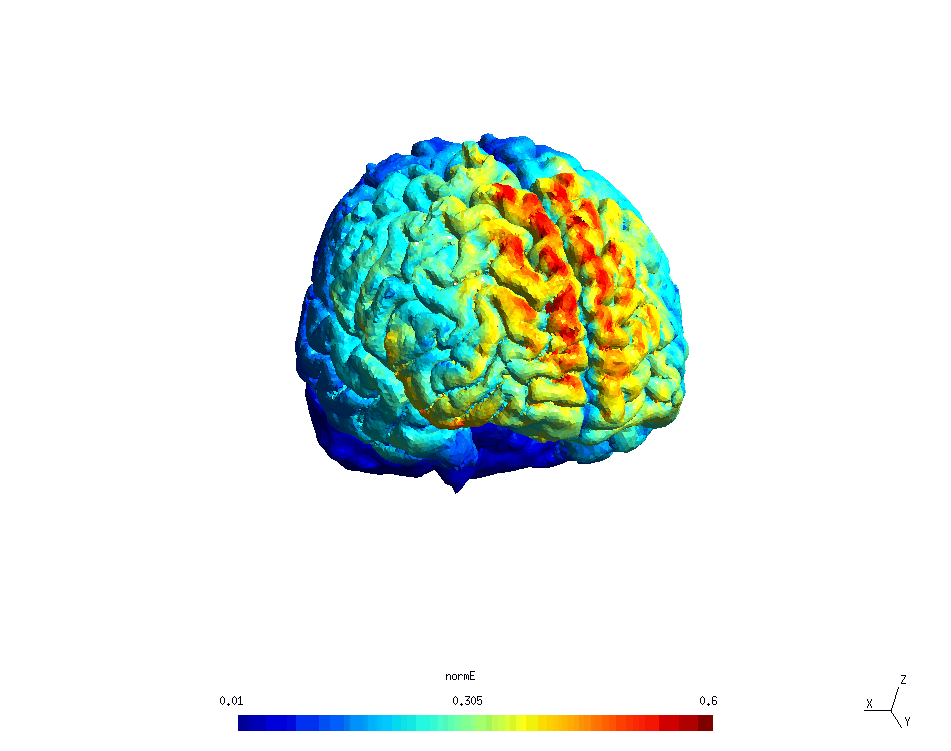

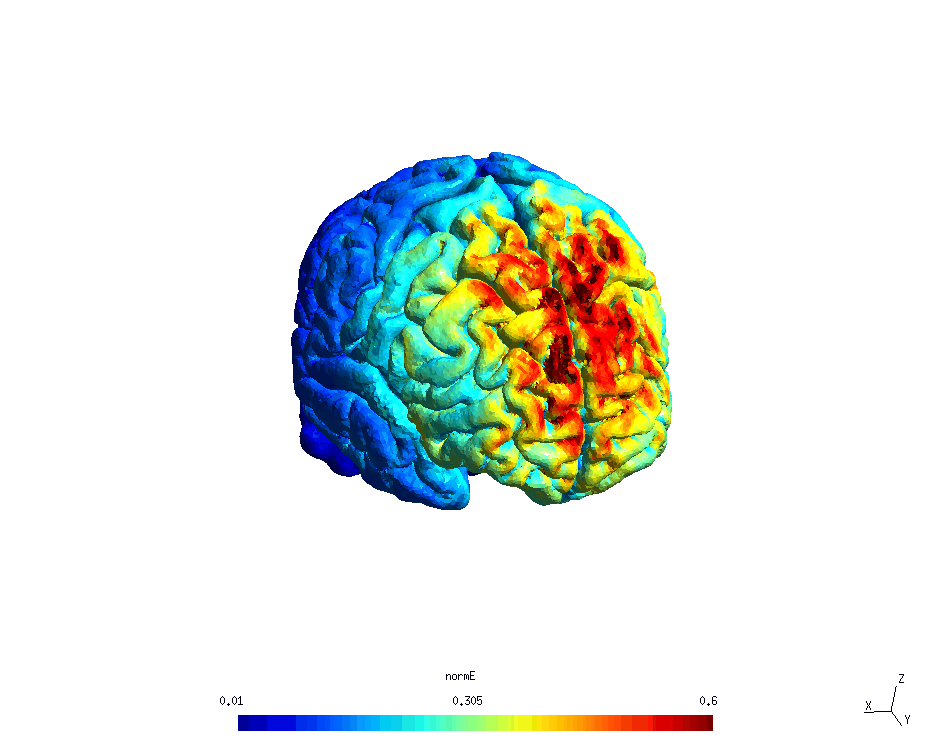

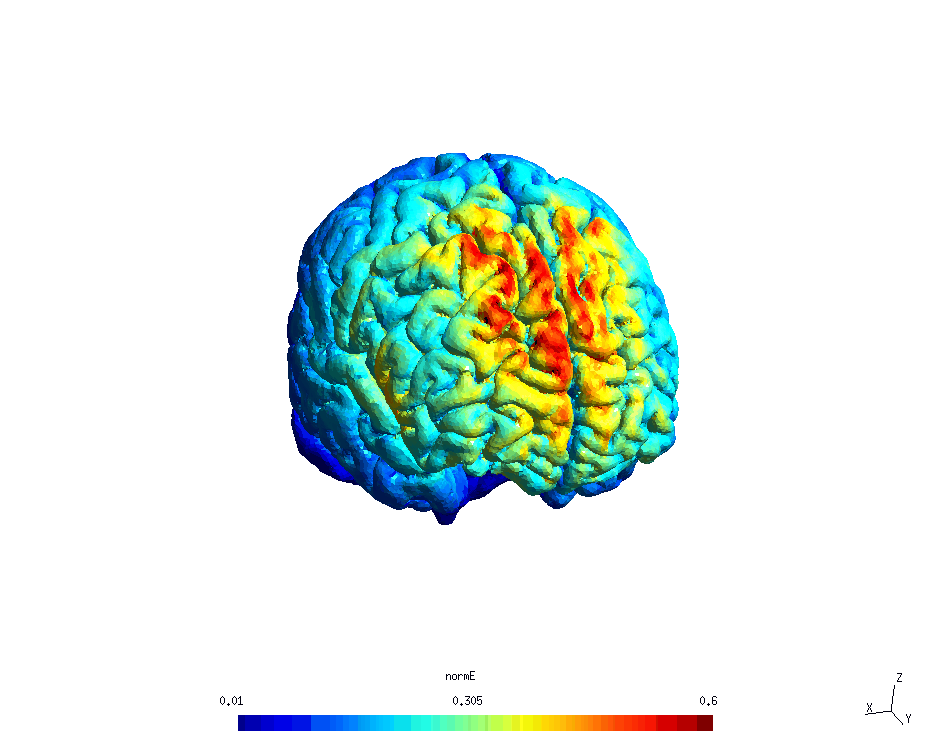

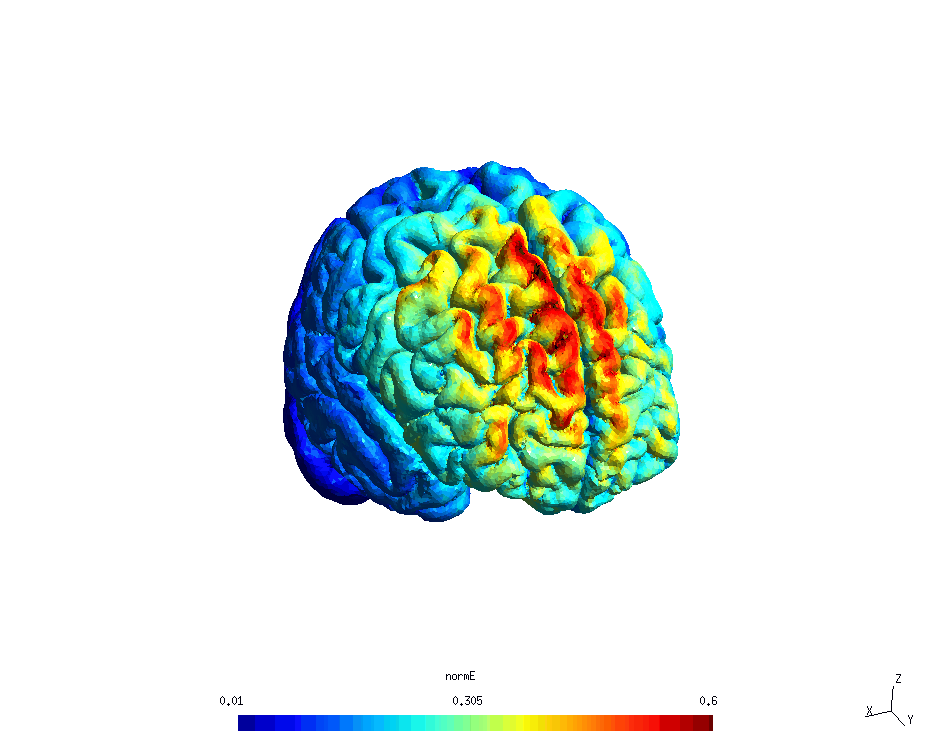

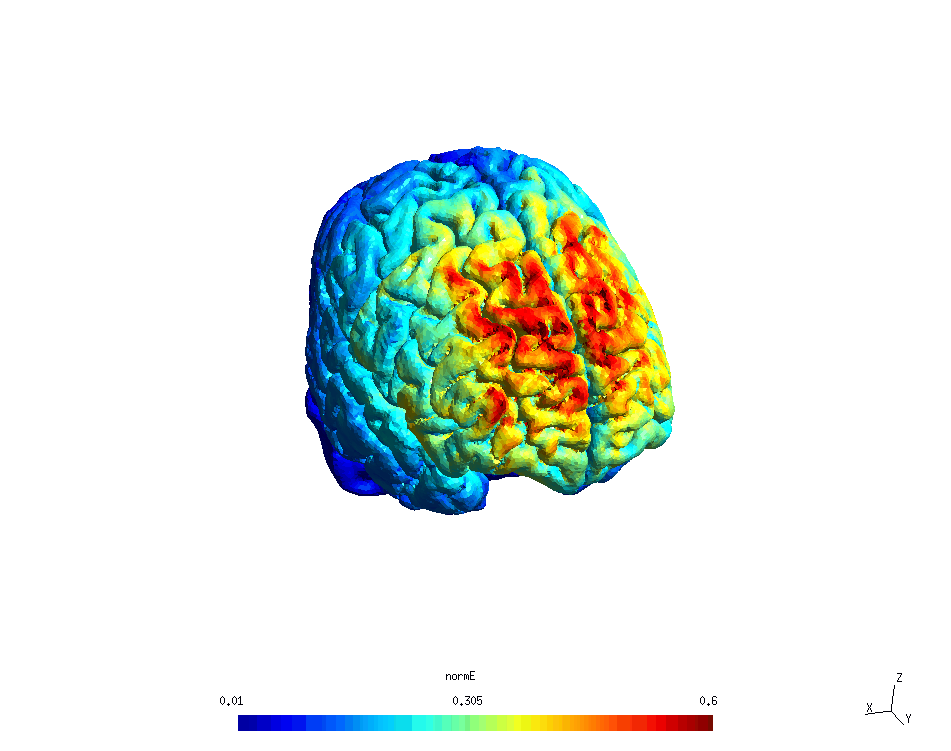

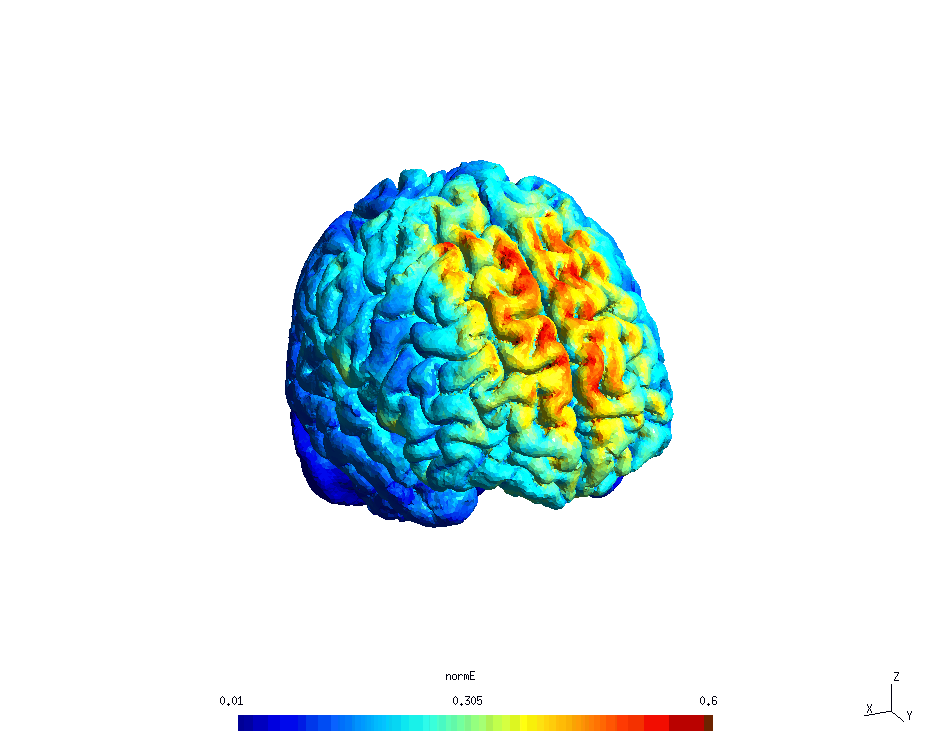

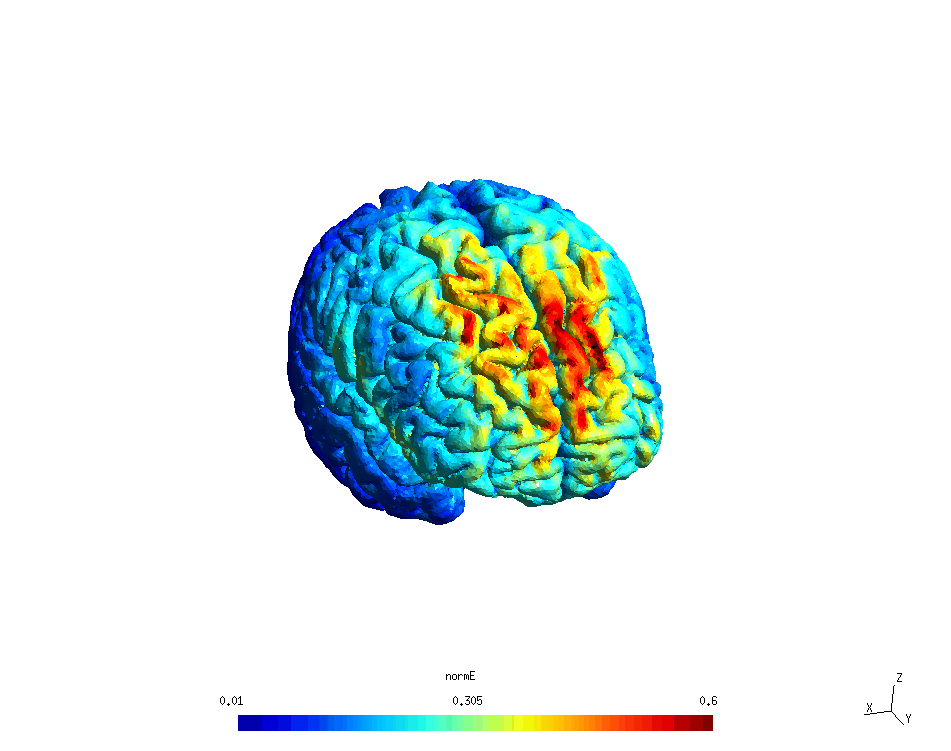

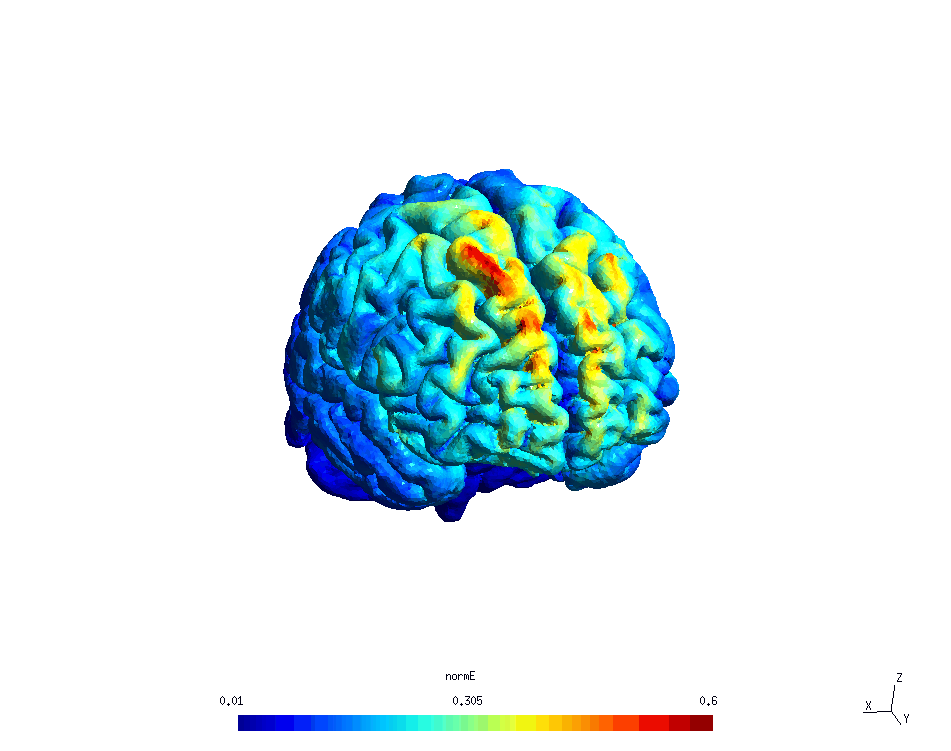

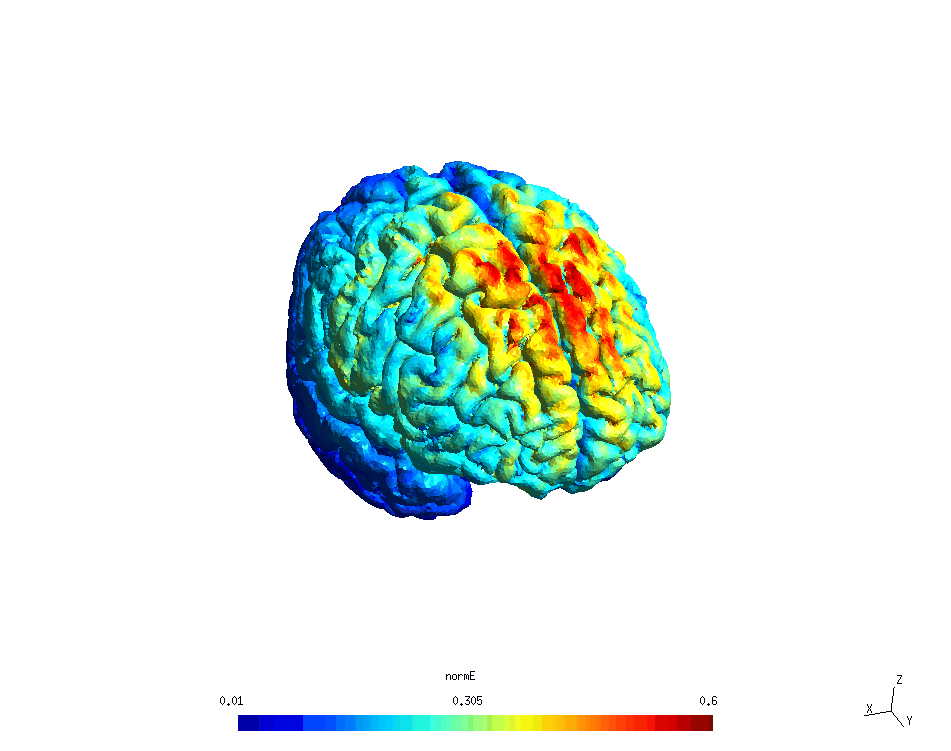
 017 018 019 020 022 023 024 025


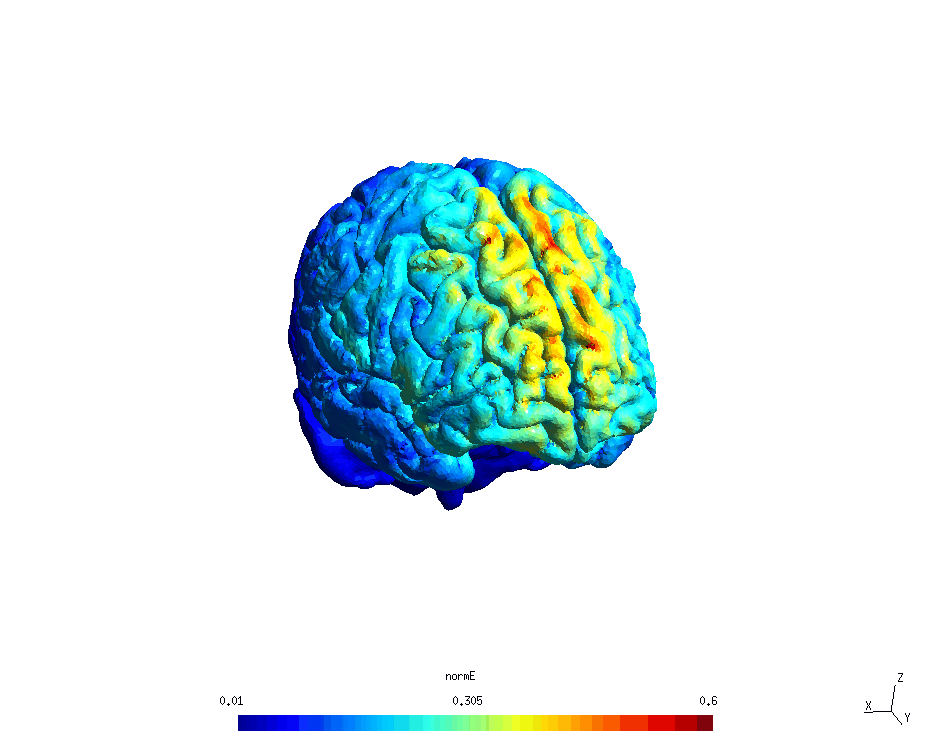

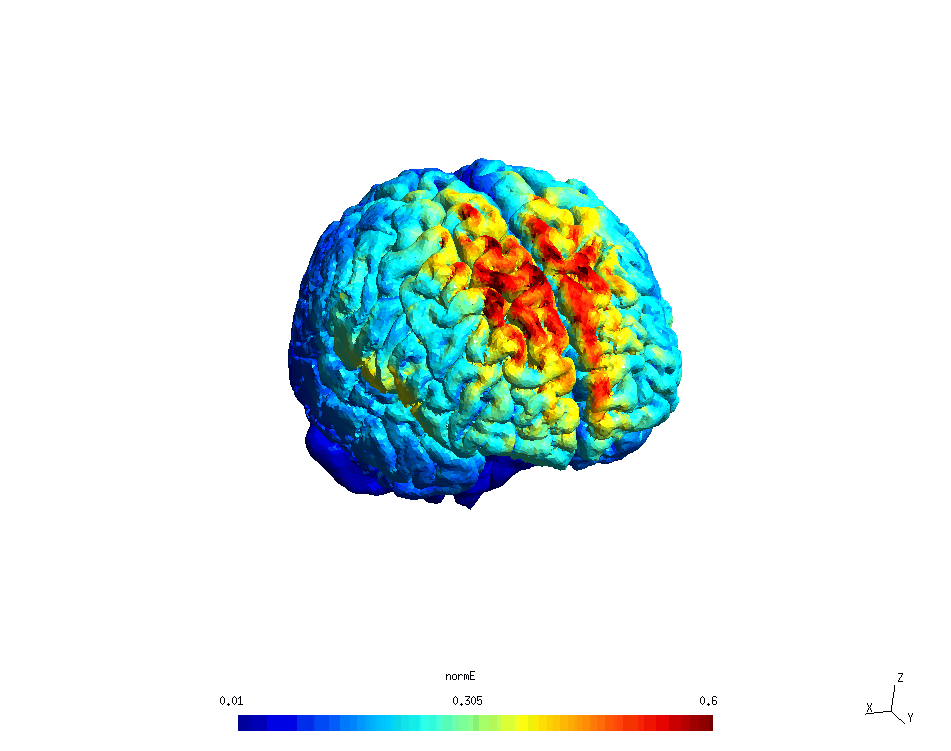

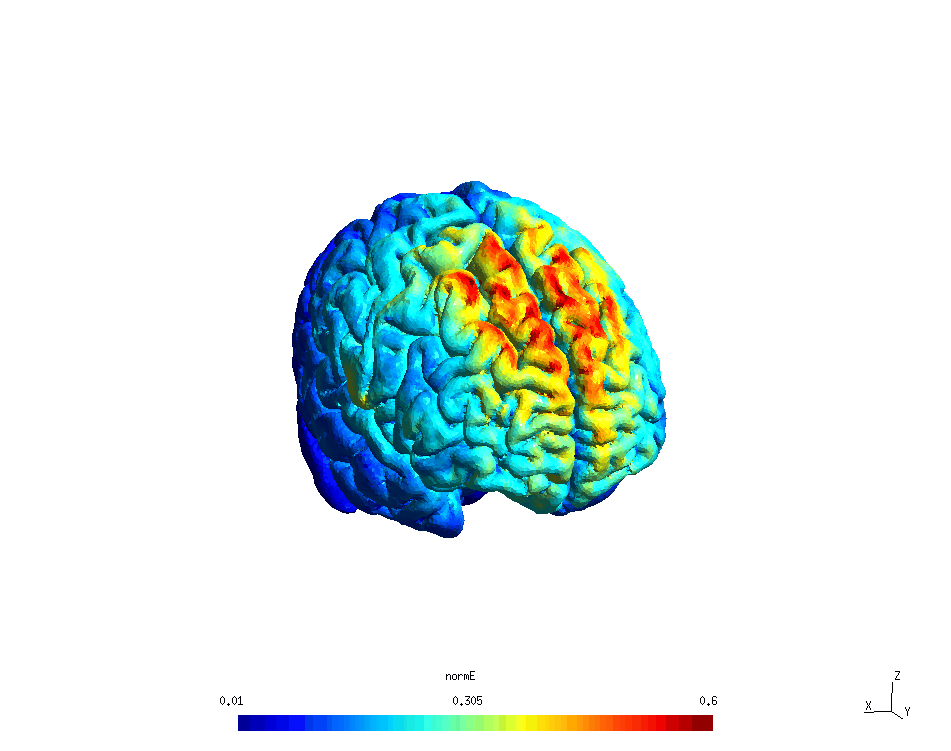

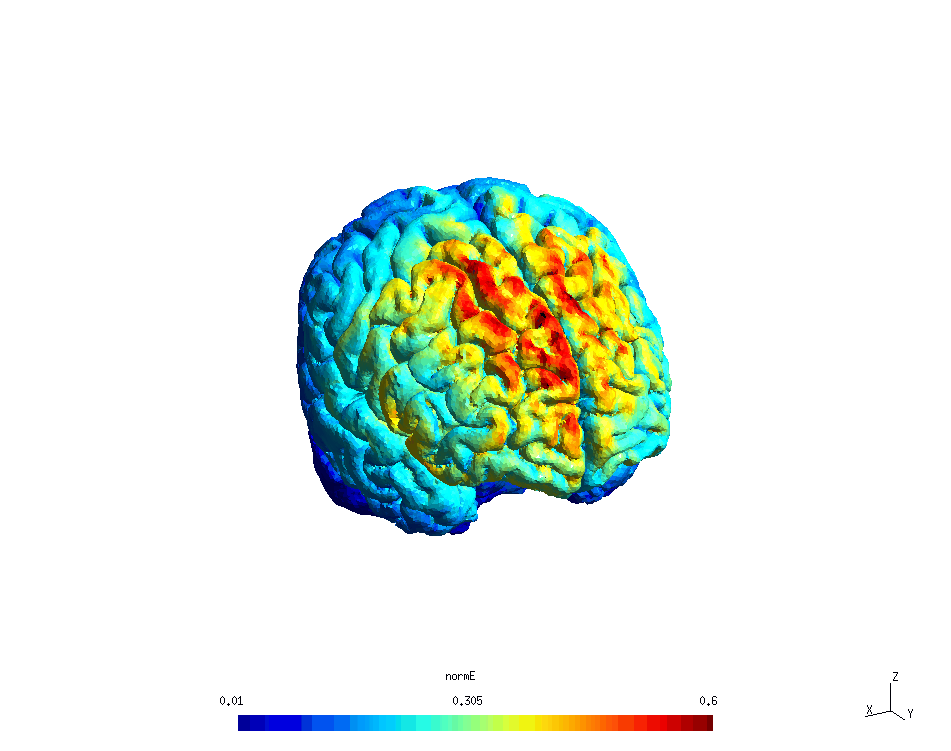

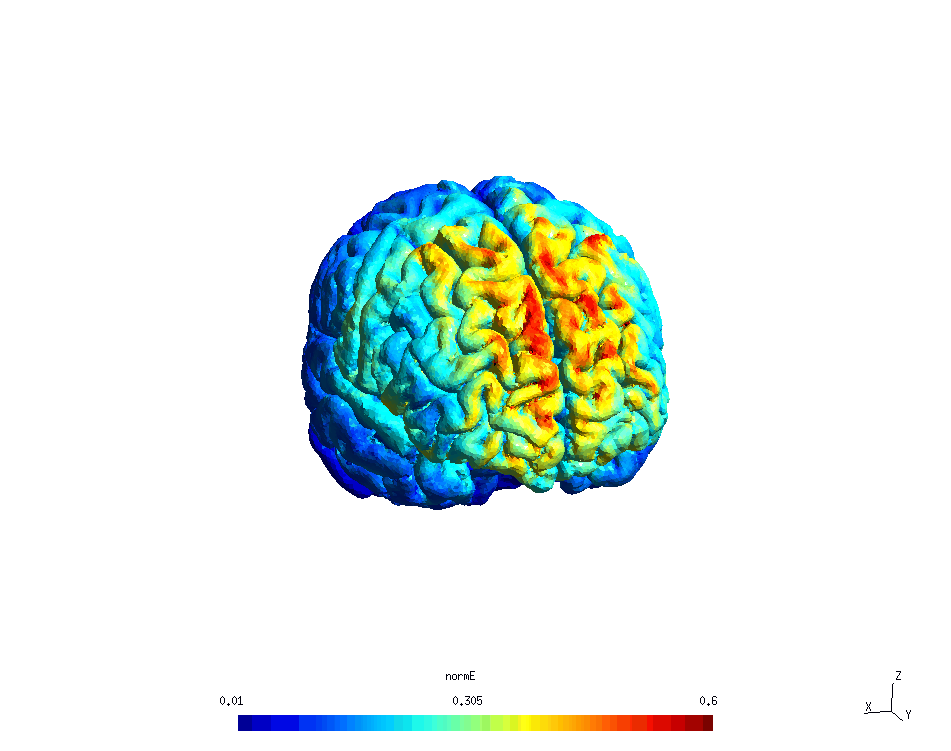

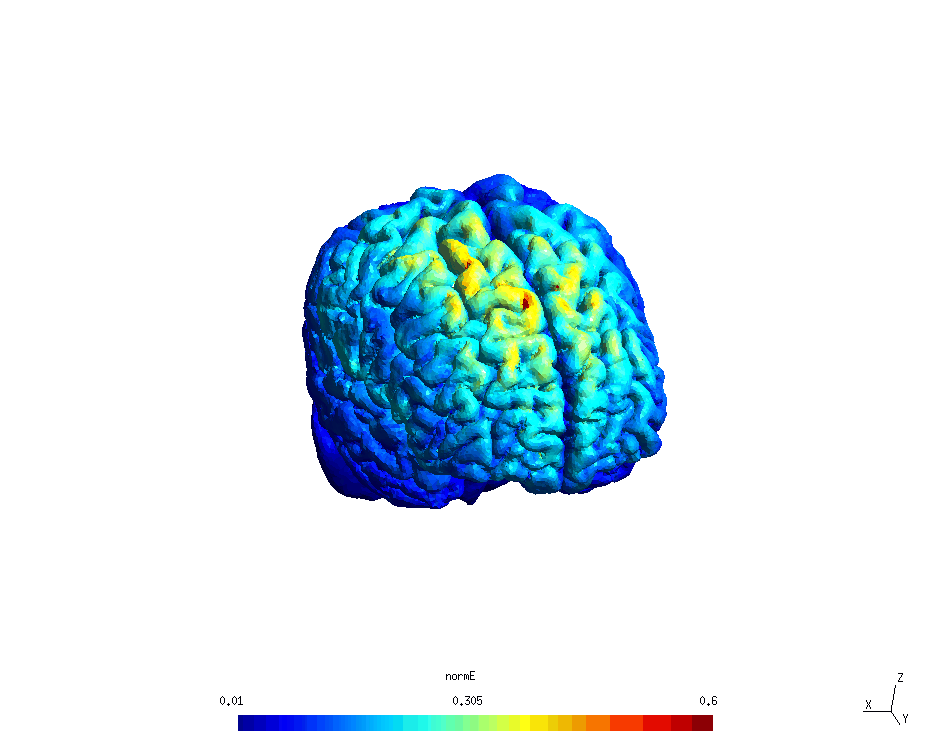

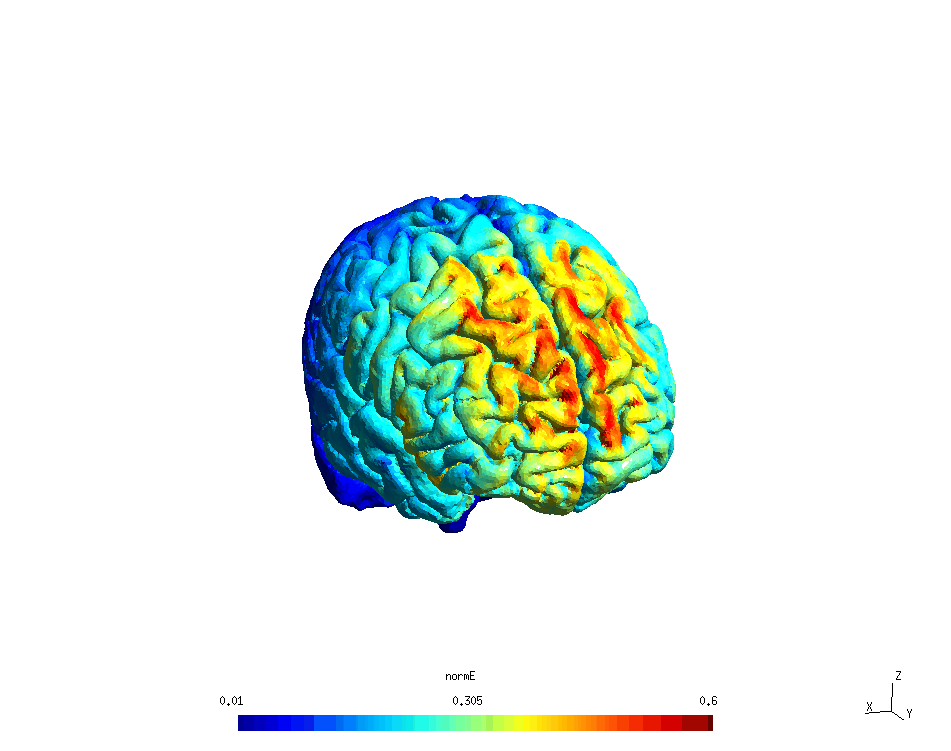

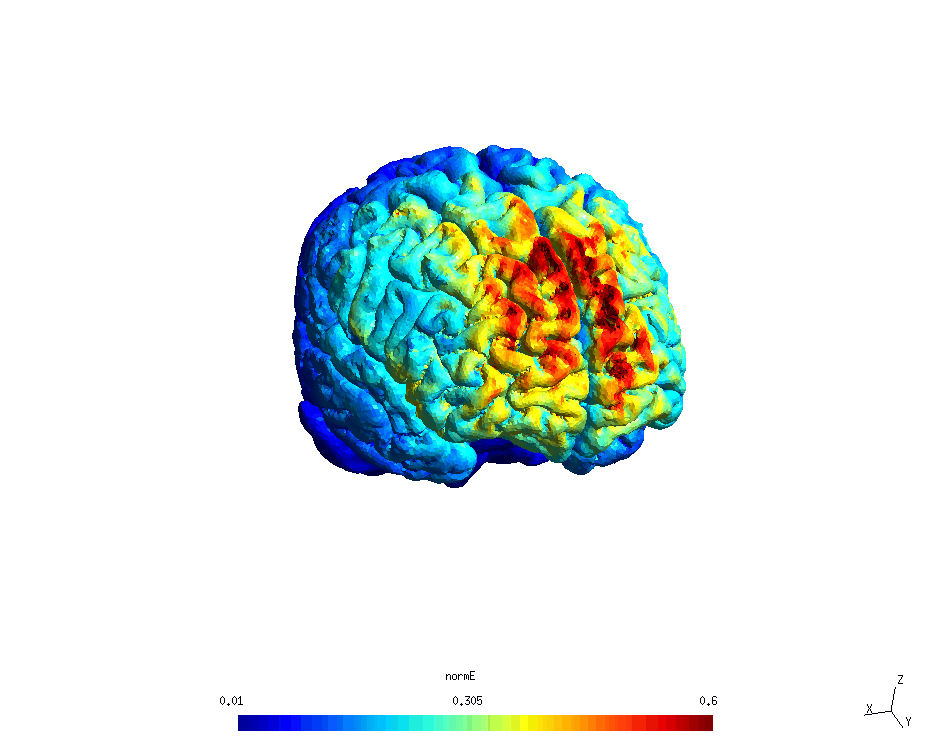


002 005 006 007 008 009 010 011 012 015


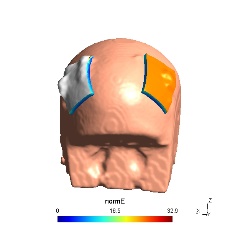

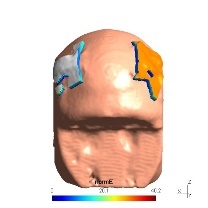

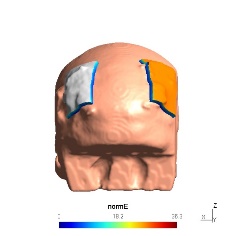

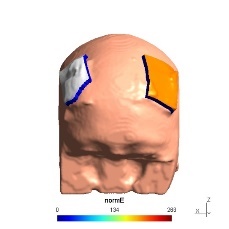

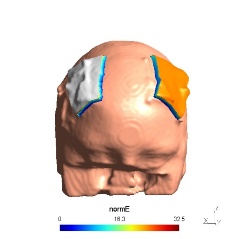

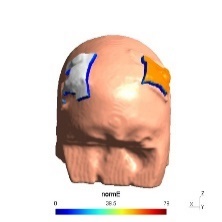

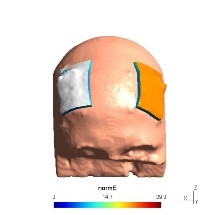

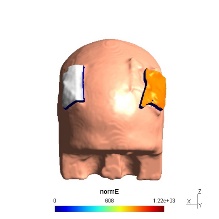

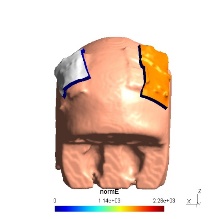

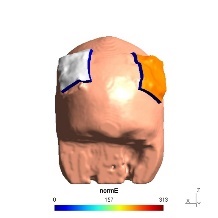


017 018 019 020 022 023 024 025


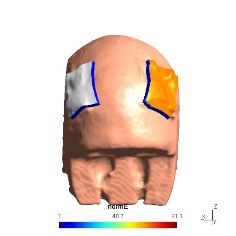

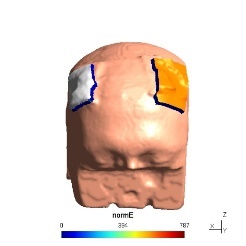

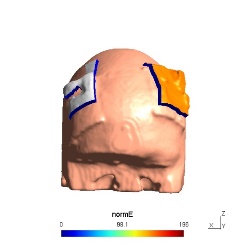

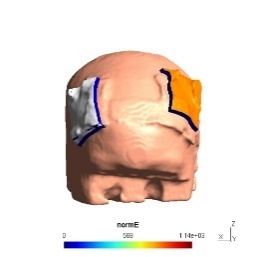

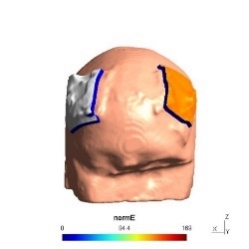

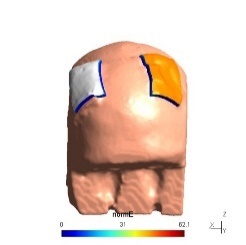

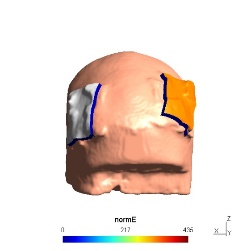

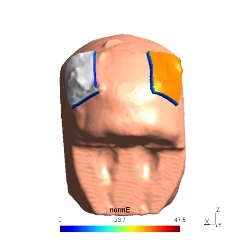


**Fig. 1** Electrical field distribution in individual heads (field intensities from min 0.01 - 0.6 V/m) and tDCS montage of individual participants. Note: the faces have been defaced to ensure the anonymity of our volunteers.

1. Methods
   1. Resting state functional MRI connectivity

Standard procedures such as brain extraction (BET) [52], binarization and segmentation into grey matter, white matter and cerebrospinal fluid using FAST [52] were performed. Linear and non-linear registration of functional MRI (fMRI) data onto the individual structural image was applied using FLIRT and FNIRT [53]. Spatial smoothing was conducted using 6 mm FWHM Gaussian kernel with high-pass temporal filtering (Gaussian-weighted, least-squares, straight line fitting with σ = 50 s). Subsequently, images were warped to the respective individual deformation template, getting normalized images in MNI space.

Analysis of rsfcMRI data was carried out in three steps; First, functional connectivity for the MRS ROI to the whole brain and within itself was analyzed to explore rsfcMRI effects close to the cathode; second, the same ROI-based rsfcMRI analysis was applied for left and right DLPFC ROIs; and third, an independent component analysis (ICA) was performed. For the ROI-based analyses (first and second), fslstats was used to extract mean of time courses of the individual ROIs (MRS ROI see Fig. 1 c and d), following cross correlation using 3dfim, z-score normalization using 3dcalc (log((1+a)/(1-a))/2 (a is the preprocessed whole brain resting state data set)) and normalization to MNI space using applywarp.

The number of activated voxels was calculated with fslmaths and a threshold of z=3. Since tDCS is generally thought to mainly act on neurons, activated voxels were only extracted from the gray matter (GM) area.

We saved the individually positioned MRS ROI coordinates over the DLPFC and converted them from native to MNI space. Seed-based connectivity calculation was carried out within the ROI and from ROI to whole brain.

The DLPFC ROIs were selected using the Sallet et al. dorsal PFC parcellation atlas, as this atlas is highly eligible for rsfcMRI analysis [54]. We selected three different ROIs from the Sallet atlas: Brodman area (BA) 46/9 dorsal, area 9 and area 10 and analyzed them both individually and together as one ROI. Third, we sequentially calculated: i) a seed-based connectivity within the ROI and additionally from the ROI to all other voxels in the brain. ii) we performed an ICA independently of the ROI analysis focussing on the default mode network (DMN) as well as on left and right fronto-parietal networks (l FPN, r FPN) as in our previous studies [25, 83]. We used the automatic dimensions estimation of MELODIC (Multivariate Exploratory Linear Optimized Decomposition into Independent Components) tool, version 3.14 and then calculated these three IC networks using dual regression for the difference between active and sham tDCS. To account for baseline activation, deltas (post-pre tDCS) were used for both conditions. A threshold value for statistical group maps was set at 3 < z < 8. At the threshold z = 3.

Effects were analysed with subtracted rsfcMR-images (post rsfcMRI data minus baseline rsfcMRI data) for both active and sham conditions applying voxel-wise nonparametric statistical contrasts (5000 permutations) using PALM alpha86 (Permutation Analysis of Linear Models; Winkler et al., 2014; Linear Models, <https://fsl.fmrib.ox.ac.uk/fsl/fslwiki/PALM/>) with two directional contrasts: brain regions with increased and reduced activity following anodal tDCS. RsfcMRI changes were considered significant at an FDR-corrected p < 0.01 (cluster size > 20 voxels).

1.
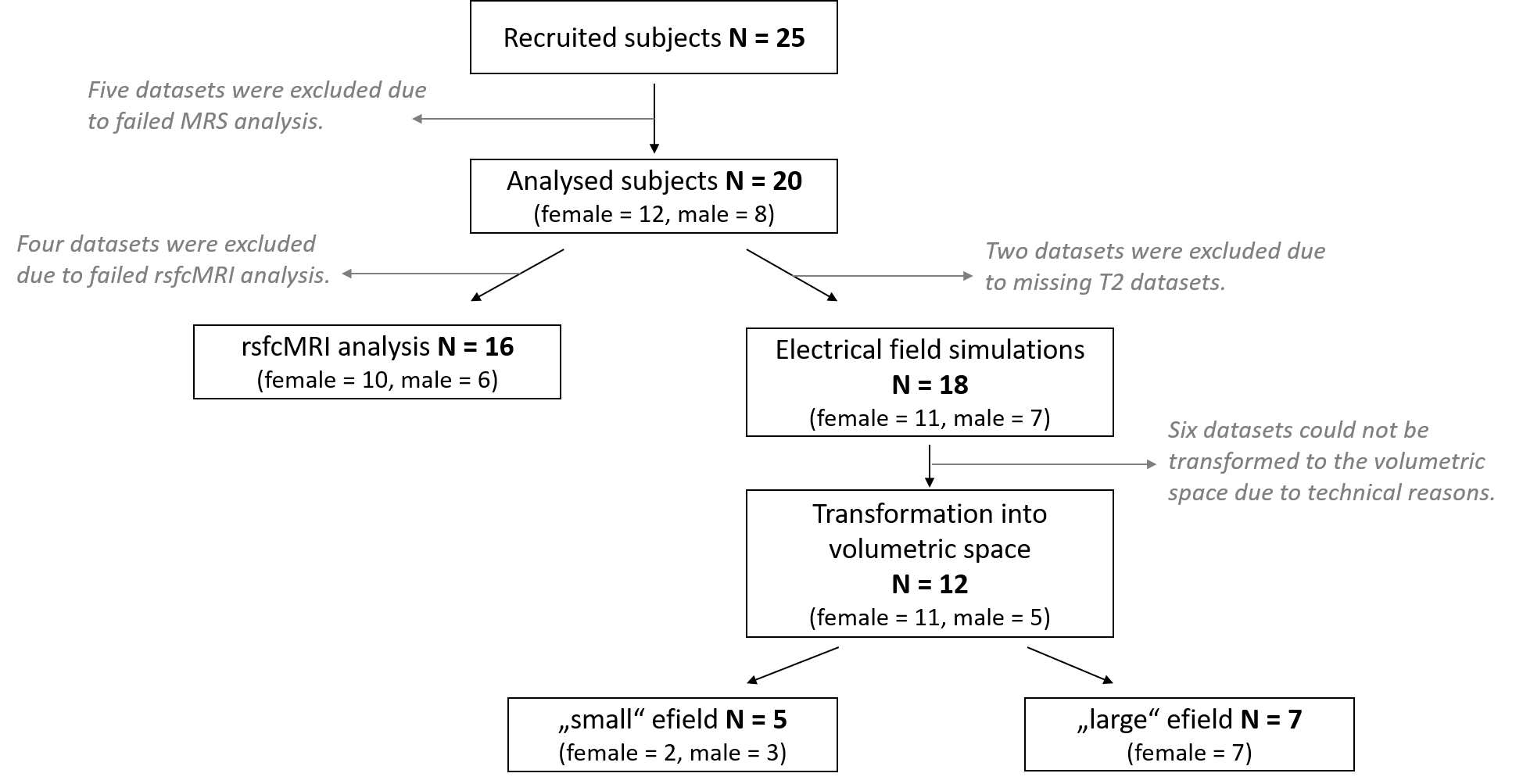
Results

**Fig. 2** Study flow chart with participants per experiment.

- 1. Affect, anxiety and side effect measures

Questionnaires were also analysed using the LMM with two time points (baseline and post stimulation). Significant results were only seen in the PANAS scores. Overall, there was a significant interaction of time*condition*PANAS score (B = 0.00 [0.00 – 0.01], *p* = .037, d = 0.34) indicating a difference of the effect of time and PANAS scores for active and sham condition. Analysis of active and sham condition separately revealed significant interaction effects of time*PANAS score in both groups (active: B = -0.00 [-0.01 – -0.00], *p* = .011, d = 0.18; sham: B = 0.08, CI = [0.00 – 0.00], *p* = .036, d = 0.18), with higher scores before the measurement (mean_active_ = 16.00±9.6; mean_sham_ = 14.95±7.1) compared to after the measurement (mean_active_ = 13.25±8; mean_sham_ = 12.15±8.6).

We separately investigated PANAS positive and negative affect scores showing a significant interaction effect of PANAS positive affect scores*condition (B = 0.01 [0.00 – 0.01], *p* = .009, d = 0.43) and PANAS positive affect scores*time (B = 0.00 [0.00 – 0.00], *p* = .036, d = 0.18), with higher scores before the measurement (mean_active_ = 28.60±7.85; mean_sham_ = 27.00±7.3) compared to after the measurement (mean_active_ = 25.75±7.6; mean_sham_ = 23.30±8.2).

The MEM with PANAS negative affect scores didn’t show any significant main effects nor interaction effects.

The STAI state questionnaire did not show any significant changes in both groups. The post measurement CRQ questionnaire showed low tDCS-induced discomfort over all participants and no significant differences between active and sham stimulation condition.

- 1. LMM of metabolite concentrations in male and female participants with and without electrical field calculations

|  |  | **Female** | | | **Male** | | |  |
| --- | --- | --- | --- | --- | --- | --- | --- | --- |
|  | *Slopes active vs sham* | *∆ Slopes* | *p* | d [CI] | *Slopes active vs sham* | *∆ Slopes* | *p* | d [CI] |
| Glu | -0.02 | 0.03 | **0.004** | 1.29  [0.41 to 2.17] | -0.00 | -0.01 | 0.440 | 0.33  [-0.50 to 1.16] |
|  | 0.01 |  |  |  | -0.01 |  |  |  |
| Glx | -0.00 | 0.001 | 0.272 | 0.56  [-0.44 to 1.56] | -0.00 | -0.002 | 0.392 | 0.87  [-2.91 to 1.16] |
|  | 0.001 |  |  |  | -0.002 |  |  |  |
| GABA | 0.00 | 0.001 | 0.449 | 0.001  [-1.72 to 1.71] | 0.00 | 0.00 | 0.327 | 0.38  [-1.15 to 0.38] |
|  | 0.001 |  |  |  | 0.00 |  |  |  |

**Table 1** Linear trajectory of metabolite concentrations during active and sham stimulation for female and male participants. Slope represents the average adjusted linear change per measurement in metabolite concentration. Cohens d represents overall change from baseline to last measurement. For Glu we observed a significant decrease during active stimulation in female participants, but not in male participants. For Glx and GABA no effects were observed. Glu = glutamate, Glx = glutamate & glutamine, GABA+ = gamma aminobutric acid (+ macromolecules). Numbers rounded to two decimal places, ∆ = difference.

|  | **Female “active” condition** | | |
| --- | --- | --- | --- |
|  | *Slope* | *p* | d [CI] |
| **Glu** |  |  |  |
| Baseline-during1 | -0.03 | **0.24** | 1.26  [-0.18 to 2.34] |
| Baseline-during2 | -0.03 | **0.004** | 1.50  [0.86 to 2.15] |
| Baseline-post | -0.02 | 0.066 | 0.84  [0.29 to 1.39] |
| During1 – during1 | -0.04 | 0.09 | 1.37  [-0.22 to 2.98] |
| During1 – post | -0.01 | 0.24 | 0.5  [-0.32 to 1.32] |
| During2 - post | 0.01 | 0.66 | 0.37  [-1.26 to 2.00] |

**Table 2** Linear change of Glu concentration in female participants by consecutively including the next latest time point from baseline during active condition. Glu = glutamate.

|  |  | **Large** | | | **Small** | | |  |
| --- | --- | --- | --- | --- | --- | --- | --- | --- |
|  | *Slopes active vs sham* | *∆ Slopes* | *p* | d [CI] | *Slopes active vs sham* | *∆ Slopes* | *p* | d [CI] |
| Glu | -0.02 | 0.02 | **0.096** | 1.12  [-0.18 to 2.44] | -0.00 | -0.01 | 0.61 | 0.29  [-0.48 to 1.43] |
|  | 0.00 |  |  |  | -0.01 |  |  |  |

**Table 3** Linear trajectory of Glu concentration during active and sham condition for large and small efield groups. Slope represents the average adjusted linear change per measurement in metabolite concentration. Cohens d represents overall change from baseline to last measurement. For Glu we observed a trend of reduction in the large efield group, but not in the small efield group. Glu = glutamate, ∆ = difference.

- 1. Effects of tDCS on resting-state functional connectivity

After active tDCS, rsfcMRI connectivity between individual MRS ROI and whole brain showed an increase within the subgenual/subcallosal cortex (at trend level; cluster-corrected at 20 voxels, Cluster: x = 2; y = 28; z = -22 (21 voxel); log-p value = 1.0; FDR-corrected, see Fig. 3). No differences were detected between active and sham tDCS for connectivity within the MRS-ROI or the ICA networks. Moreover, no correlation between changes in functional connectivity and metabolite levels was observed.


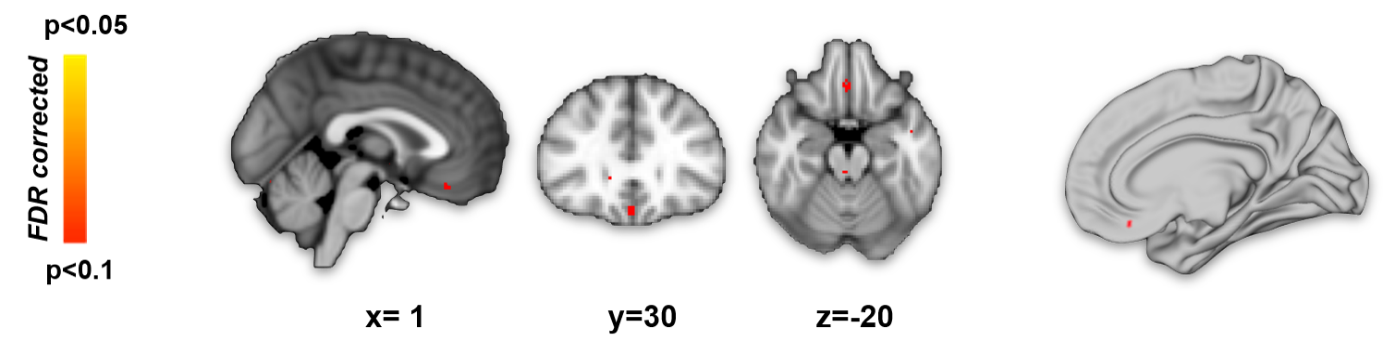


**Fig. 3** ROI-based rsfcMRI of the individual MRS voxel to the whole brain indicates increased connectivity from the MRS-ROI to the subgenual / subcallosal cortex after active stimulation. Results are FDR-corrected log-p values (threshold between max. 1.3 (p<0.05) min. 1 (p<0.1)). Colored voxels represent the following contrasts: yellow = whole brain delta (post > pre) active tDCS > whole brain delta (post > pre) sham tDCS. Cluster: x = 2; y = 28; z = -22 (21 voxel).

1. **
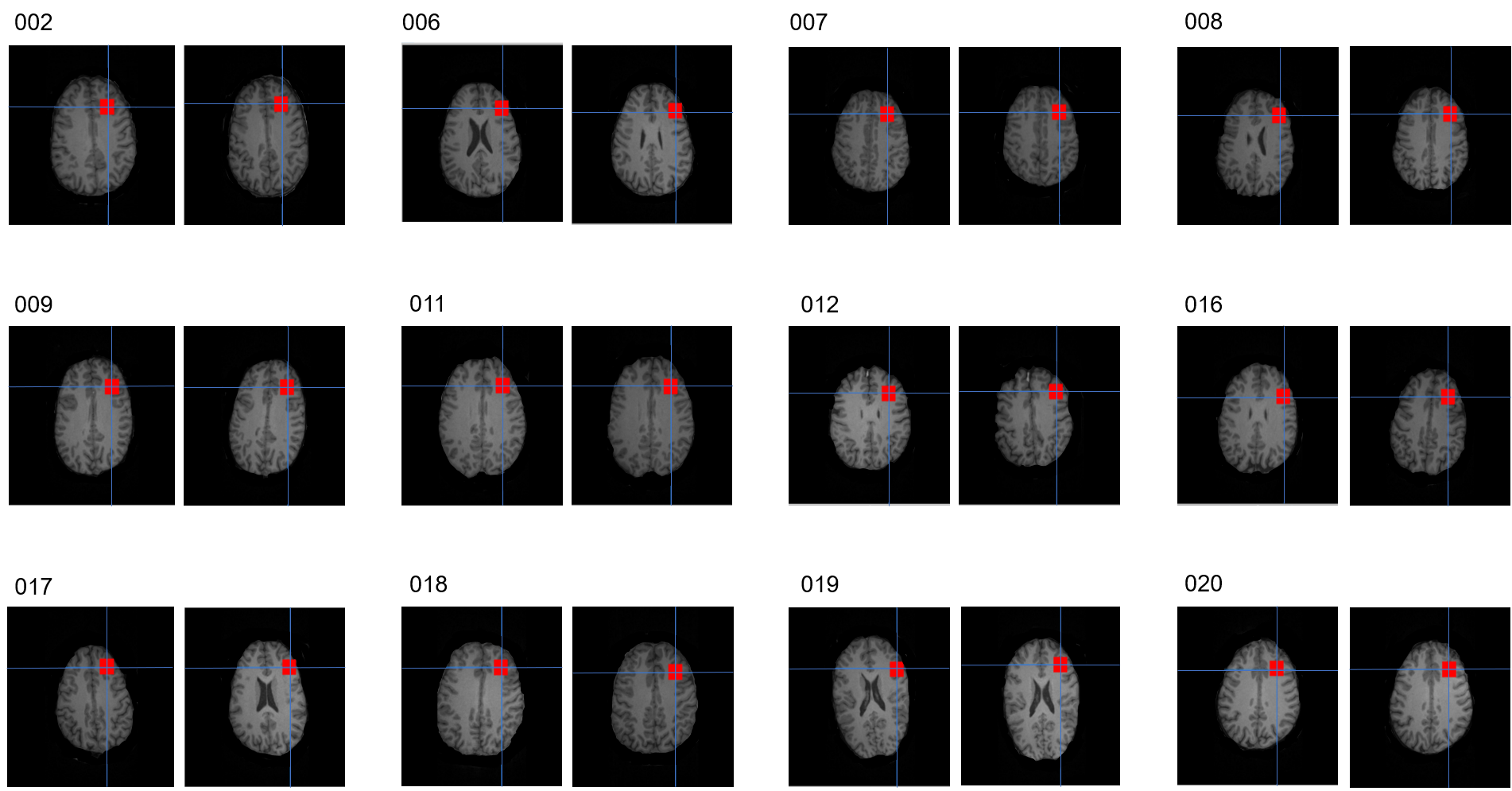
**Further information

**Fig. 4** Example of ROI location in the brain (left = active stimulation; right = sham stimulation

- 1. GABA and Glx data was analysed using Gannet 3.0.

Gannet 3.0 Output (<http://www.gabamrs.com>)


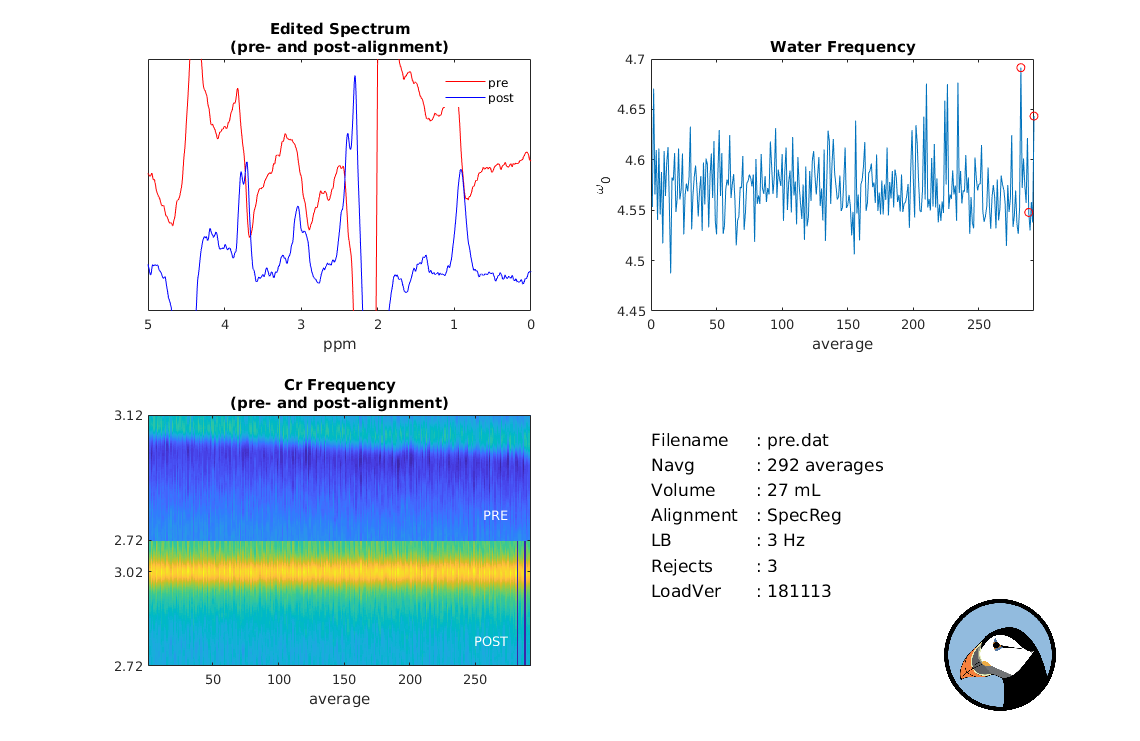


**Fig. 45** GannetLoad Output: In the upper left it shows the GABAedited difference spectrum which is the most important part of this output. This graph shows the subtracted MRS spectrum before phase and frequency correction (in red) and after correction (in blue). The upper right part shows the maximum of the spectrum, usually the residual water signal. On the lower left side, the creatine signal over the time of the experiment is shown. The y-axis depicts the frequency in ppm whereas the lighter color shows higher signal intensities. As this plot shows the creatine signal there should be a light-colored stripe running through the picture at 3 ppm. Again, pre and post show the creatine signal before and after phase and frequency correction.


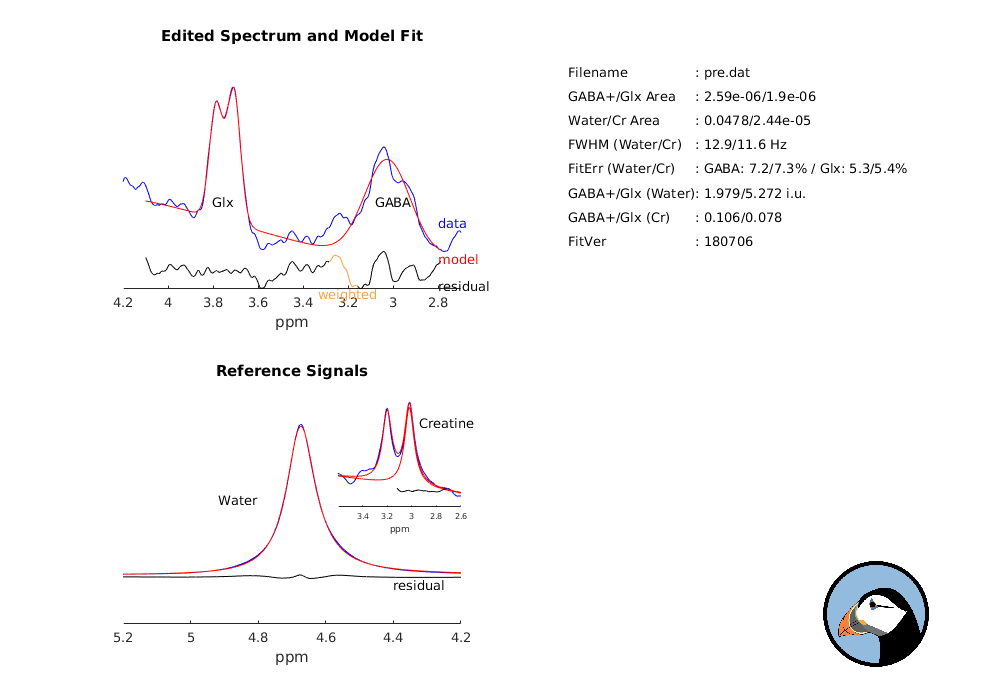


**Fig. 6** GannetFit Output: The second output of Gannet shows on the upper left the model fit of the GABA+ signal. In blue the GABA+ edited signal is shown and in red the best model fit by calculating a Gaussian model. The residual of the model and the actual GABA+ signal is shown at the bottom in black. The lower left part shows a graph with the reference signals against which GABA+ is quantified.


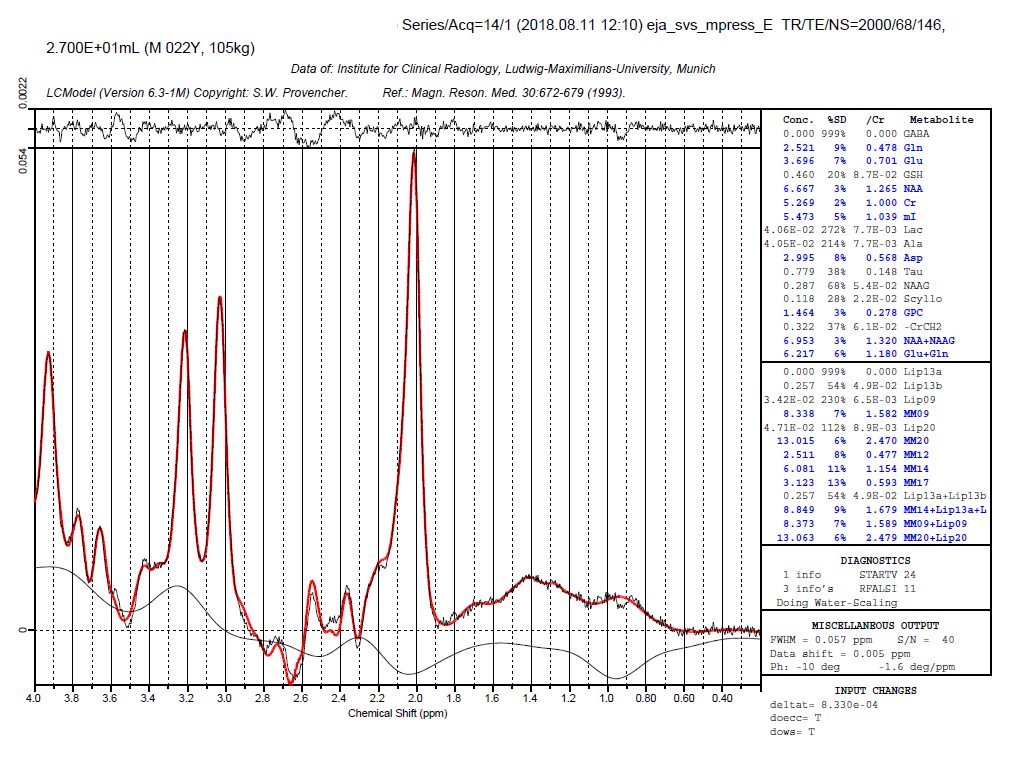


**Fig. 7** LcModel example Output of MRS MEGA-PRESS data using OFF-Spectrum in LcModel (http://s-provencher.com/lcmodel.shtml) for Glu quantification. Only data with %SD (Cramér-Rao lower bounds) < 20 % was used for further analysis. The plot shows the real part of the data (thin black line) and the LCModel fit to the data (red line). At the top the Residuals are plotted, showing the data minus the fit of the data.

- 1. Metabolite basis-set and processing steps

The LcModel basis set used for metabolite quantification includes 22 metabolite spectra: acetate (Ace), alanine (Ala), aspartate (Asp), choline (Ch), phosphocholine (PCh), glycero-PC (GPC), Cr, phospho-Cr (PCr), gamma aminobutric acid (GABA), glucose (Glc), glutamine (Gln), glutamate (Glu), glutathione (GSH), glycine (Gly), lactate (Lac), myo-inositol (mI), scyllo-inositol (sI), N-acetyl-Asp (NAA), NAA-glutamate (NAAG), phosphoethanolamine (PE), taurine (Tau), and threonine (Thr).

LcModel analyses an in vivo spectrum (.rda data) as a linear combination of the model in vitro spectrum. Following steps are included: 1. T2 broadening and small frequency shifts in case of referencing errors. 2. Pooling of field inhomogeneities, eddy currents and frequency drifts by using fast Fourier Transformation. 3. Baseline, zero – and first-order phase corrections for the in vivo spectrum. 4. A nonlinear least-squares analysis calculates metabolite concentrations and their uncertainties.

For GABA+ quantification we used the GABA+ specified analysis software Gannet. Processing steps using .rda data include following steps: 1. Time-domain frequency-and-phase correction using spectral correction. 2. Exponential apodization function (line broadening). 3. Fast Fourier Transformation. 4. Frequency and phase correction (of edit-OFF.rda to edit-ON.rda) based upon fitting of the Cho and Cr signals. 5. Subtraction to generate the edited difference spectrum (and extraction of edit-OFF spectrum).”
